# School achievement as a predictor of depression and self-harm in adolescence: linked education and health record study

**DOI:** 10.1192/bjp.2017.69

**Published:** 2018-03-06

**Authors:** Muhammad A Rahman, Charlotte Todd, Ann John, Jacinta Tan, Michael Kerr, Robert Potter, Jonathan Kennedy, Frances Rice, Sinead Brophy

**Affiliations:** 1 FARR Institute, School of Medicine, Swansea University, Swansea; 2 Institute of Life Sciences, School of Medicine, Swansea University, Swansea; 3 Division of Psychological Medicine and Clinical Neurosciences, Cardiff University, Cardiff; 4 Cwm Taf Health University Health Board and Institute of Psychological Medicine and Clinical Neuroscience, Cardiff University School of Medicine, Cardiff; 5 FARR Institute, School of Medicine, Swansea University, Swansea; 6 Division of Psychological Medicine and Clinical Neurosciences, Cardiff University, Cardiff; 7 FARR Institute, School of Medicine, Swansea University, Swansea

## Abstract

**Background:**

Mental disorders in children and adolescents have an impact on educational attainment.

**Aims:**

To examine the temporal association between attainment in education and subsequent diagnosis of depression or self-harm in the teenage years.

**Method:**

General practitioner, hospital and education records of young people in Wales between 1999 and 2014 were linked and analysed using Cox regression.

**Results:**

Linked records were available for 652 903 young people and of these 33 498 (5.1%) developed depression and 15 946 (2.4%) self-harmed after the age of 12 but before the age of 20. Young people who developed depression over the study period were more likely to have achieved key stage 1 (age 7 years) but not key stage 2 (age 11) (hazard ratio (HR) = 0.79, 95% CI 0.74–0.84) milestones, indicating that they were declining in academic attainment during primary school. Conversely, those who self-harmed were achieving as well as those who did not self-harm in primary school, but showed a severe decline in their attainment during secondary school (HR = 0.72, 95% CI 0.68–0.78).

**Conclusions:**

Long-term declining educational attainment in primary and secondary school was associated with development of depression in the teenage years. Self-harm was associated with declining educational attainment during secondary school only. Incorporating information on academic decline with other known risk factors for depression/self-harm (for example stressful life events, parental mental health problems) may improve risk profiling methods.

The prevalence of mental disorders in children and adolescents is estimated at 10–20% worldwide^1^ and mostly goes unrecognised and untreated. Moreover, 50% of adult mental disorders starts during or before adolescence^2^^,^^3^ and therefore, identification of children at risk and prevention or early treatment are essential in order to reduce the later burden of mental illness. Depression and self-harm are the leading causes of disability in adolescents and young adults, and the largest contributor to years lived with disability.^4^^,^^5^ Recent reports in the UK suggest that service demand among young people in this age group is increasing, with nearly 90% of mental health professionals noting an increase in referrals of both routine and emergency presentations over recent years.^6^ Recent media reports suggest the number of children and young people attending accident and emergency departments with mental health conditions has more than doubled since 2009.^7^ In addition, the World Health Organization (WHO) survey of health behaviour in school-age children finds UK teenagers among the least happy in the world.^8^

Reflecting the concern associated with the social and economic impact of poor mental health, early identification and prevention is a major aim for many governments and health agencies (for example the WHO mental health strategy). For early identification and prevention strategies to be effective they need to be informed by an understanding of how the disorder first develops. Although it is recognised that mental health disorders involve multiple causal factors (social, psychological, family, genetic), several studies have identified low levels of educational attainment as an important association with depression and self-harm during childhood and adolescence.^9^^–^^11^ The link between education and mental health is generally believed to be complex and reciprocal, with academic success giving a strong subjective sense of children feeling good about themselves and being linked to higher levels of well-being in adulthood^12^ and poor academic attainment been identified as co-occurring with symptoms. However, studies exploring this issue have been limited in the extent to which they take coexisting problems into account^13^ and fewer studies have explored these associations over time.^8^ A systematic review examining such longitudinal associations found that depression was associated with poor later school attainment and that associations between depression and school failure were stronger for girls than boys.^14^ Authors highlighted a need for further longitudinal research to give greater insight into these associations. Indeed, the measures of mental health and education used vary widely in the literature and the extent to which prior attainment influences the development of both internalising (depression/self-harm) and externalising problems (conduct disorder, attention-deficit disorder) in adolescence is also not yet fully understood. Understanding the strength of these associations through childhood and adolescence has important policy and practice implications; particularly given the UK has high levels of inequalities in educational attainment among children.^15^ It may also help inform decisions about which individuals should be targeted for early intervention given that prevention and early intervention programmes for depression show larger effects for indicated (that is targeted at those at increased risk of developing depression) than universal programmes.^16^ Although early detection and treatment^17^ is known to be beneficial, it is still the case that most depression in adolescence is untreated. The National Institute for Health and Care Excellence guidelines suggest risk profiling in primary care settings including schools.^18^ In this study, we set out to examine if educational attainment could be one way of identifying individuals more prone to depression and self-harm. This study aims to examine the temporal link between prior educational attainment and later diagnosis of depression or self-harm. This comparison will examine to what extent educational attainment can help identify individuals with diagnosed depression and reported self-harm after adjusting for socioeconomic deprivation and behavioural problems and their associated medications.

## Method

### Study population and data-sets

The population we selected from consisted of all children and young people (aged 5–20) born or living in Wales who had at least 1 year in education at school in Wales between the years 1990 to 2014. Education records were linked with mortality data, hospital admissions data and general practice records. The linkage and hosting of this data were through the SAIL (Secure Anonymised Information Linkage) databank.^19^^,^^20^ The SAIL databank anonymously record-links routinely collected data held in health and social care data-sets at the Centre for Improvement in Population Health through E-records Research (CIPHER), Swansea University, UK, which is part of the Farr Institute (http://www.farrinstitute.org/). For each data-set within the SAIL databank, an individual is assigned an anonymised linking field (ALF_E), based on their names, addresses or National Health Service (NHS) numbers, which is employed to link across data-sets. All data within the SAIL gateway is treated in accordance with the Data Protection Act 1998. To date, the SAIL databank incorporates over 10 billion records from multiple health and social care events and at the time of analysis, received data from 70% (328/468) of the general practitioner (GP) practices in Wales and all hospital admissions. The education database is available for all children in school between the years 2005 and 2014. Therefore, the study population consisted of children with a GP record (60% of all children in Wales) and linkage to the educational data-set (for example children of school age between the years 2005 and 2014).

### Statistical analysis

The SAIL databank was queried using IBM DB2 9.7 SQL. Statistical analyses were conducted using Stata version 13. Exposure was attainment in education at the key stages within the education data-set. Mental health as an outcome was examined through looking at diagnosis and symptom codes for depression and self-harm in GP data in those aged ≥12,^21^^,^^22^ and confounders examined included a diagnosis of attention-deficit hyperactivity disorder (ADHD) or conduct disorder or intellectual disability (also known at learning disability in UK health services), prescriptions of hypnotics, stimulants and gender (captured from the GP data-set) and socioeconomic status in primary school as measured by free school meal eligibility captured in the educational data-set (see Supplementary Table 1, available at https://doi.org/10.1192/bjp.2017.96 for the Read codes and ICD-10 codes used to identify diagnosis).

The diagnosis of depression included: single or recurrent major depressive episode and symptom codes for depression such as depressed mood. The diagnosis for self-harm included: self-inflicted, intentional codes and injury undetermined intent codes (see Supplementary File 1). Undetermined intent codes are included as this is what is recommended in the literature because of some suicides being recorded as ‘undetermined intent’.^23^ In this study 3% of the self-harm cases considered were of ‘undetermined intent’. A sensitivity analysis involved fitting separate models for males and females (Supplementary File 2). Results were the same as for the full data-set and we therefore report results from the full sample here.

Outcomes assessed were time to event between the ages of 12 and 20 in the in-patients' data-set and visits to the GP. Analysis was conducted for each outcome separately (Table 1) and Cox regression analysis was used to examine the relationship between academic attainment and outcomes adjusted for confounders (i.e. gender, deprivation, previous attainment, intellectual difficulties and diagnosis of behavioural problems). The follow-up was calculated as time from age 12 to the outcome of interest or censored at date of death or date of end of study. Only children with educational records at the time of interest were analysed and no imputation was undertaken to estimate missing data.

**Table 1:** Cox regression analysis – exposures and outcomes

	Exposure	Outcome
1	KS1 (age 7) and KS2 (age 11)	Depression at age 12–20 Self-harm at age 12–20
2	KS1(age 7), KS2, (age 11) and KS3 (age 14)	Depression at age 14–20 Self-harm at age 14–20
3	KS1 (age 7), KS2 (age 11), KS3 (age 14), KS4 (age 16)	Depression at age 16–20 Self-harm at age 16–20
4	Depression age 12–14 Self-harm age 12–14	KS3/4 KS3/4

KS, key stage.

GP data in the UK are coded using Read codes, which contain some 300 000 codes for symptoms, diagnosis, treatment and management. Data within the hospital admission system are recorded using ICD-10 codes.^24^ The Read and ICD-10 codes used to identify mental health problem and drugs can be found in Supplementary File 1. The age at depression or self-harm was assumed to be age at first mention in the healthcare record. Individuals with a pre-existing diagnosis of depression or self-harm in primary school were excluded (see below) as we were using educational attainment as the exposure to predict future mental health conditions.

### Ethical approval

The study design uses anonymised data and therefore the need for ethical approval was waived by the approving institutional review board. The independent Information Governance Review Panel, which contains members from the UK National Health Service Research Ethics Service, approved the study.

## Results

There were approximately 1 million children born and subsequently living in Wales between 1 January 1990 and 31 December 2014 (male: 538 181; female: 519 191; unknown gender: 21). Of these, 829 590 could be linked to the education data-set (pre-16 years educational attainment data-set) that was available for children in school between 2005 and 2014. The education database contains the school attainment results. This data-set contains results for; key stage 1 (KS1) that covers national tests in mathematics and English/Welsh language at age 6/7; key stage 2 (KS2) that covers national tests in the core subjects of mathematics, English/Welsh and science at ages 10/11; key stage 3 (KS3) that covers national tests at ages 13/14, including both core and non-core subjects; and key stage 4 (KS4) that covers a range of subjects at age 15/16.

In this linked data-set of 829 590, there were 627 423 (76%) children who were over the age of 5 years (male: 319 839; female: 307 584) and had at least one result in KS1 through to KS4. An individual was considered to have achieved their key stage if they passed mathematics and English/Welsh to the accepted national curriculum level. If they did not achieve the accepted level in the core subjects (mathematics or language (English/Welsh)) they were assigned an overall ‘not achieved’.

### Depression

There were 33 498 individuals who had a diagnosis/symptoms of depression in adolescence and an additional 605 children who were excluded from the analysis who had depression diagnosed in primary school. These children (with a pre-existing diagnosis of depression) were excluded as we were using educational attainment as the exposure to predict future mental health conditions. Girls were more likely to be diagnosed with depression than boys (7.49 *v.* 3.27%, difference: 4.2%, 95% CI 4.1–4.3) and were more likely to have been prescribed an antidepressant drug (6.92 *v.* 3.3%, difference: 3.6%, 95% CI 3.5–3.7) (Table 2). The crude hazard ratio (HR) of developing depression (years of follow-up 11 404 720) if not achieving KS2 (age 11) was 1.25 (95% CI 1.21–1.29) and the adjusted HR was 1.26 (95% CI 1.18–1.34) (Table 3). Adjusting for deprivation (free school meal status), ADHD, learning difficulties, conduct disorder, prescription of hypnotic or stimulant in primary school and gender it was found that those who were achieving at KS1 (aged 7) but not at KS2, the end of primary school (i.e. declining in educational attainment during primary school), were more likely to have depression in adolescence. This decline continues, with young people who go on to be diagnosed with depression after the age of 14 being 38% less likely to have achieved their KS3 (before diagnosed with depression) and 50% less likely to have achieved KS4 (when diagnosed with depression after KS4) than those who are not diagnosed with depression (Table 4). Thus, children were declining in school long before a diagnosis of depression. Those with depression in the months before or at the time of their exams/assessment were also 40% less likely to achieve their key stage than those without depression. Deprivation, female gender, conduct disorder (diagnosed in primary school) were also associated with higher risk of future depression and intellectual disability was associated with lower rates of diagnosed depression (Table 3 and Supplementary File 2).

**Table 2:** Key stage (KS) achievement at KS1 (age 7), KS2 (age 11), KS3 (age 14) and KS4 (age 16) (male and female), free school meal eligibility and mental health problem and drug rate

	Male	Female
Exposure variables, % (*n*/*N*)		
Age 7 achieved	77.5 (129 884/167 599)	86.10 (136 685/158 751)
Age 11 achieved	73.1 (128 587/175 924)	80.92 (135 799/167 829)
Age 14 achieved	57.9 (108 881/188 172)	67 46 (121 859/180 637)
Age 16 achieved	37.7 (54 620/144 795)	42.5 (61 162/143 879)
Free school meals at age 7	19.4 (32 452/167 496)	19.8 (31 427/158 644)
Free school meals at age 11	18.7 (32 921/175 860)	19.1 (32 023/167 773)
Free school meals at age 14	17.2 (32 411/188 133)	17.2 (31 093/180 606)
Free school meals at age 16	13.9 (20 018/144 222)	14.0 (20 141/143 349)
*Outcome variables*		
Depression, % (*n*/*N*)	3.27 (10 458/319 839)	7.49 (23 040/307 584)
Age, mean (s.d)	19.17 (3.18)	18.72 (2.91)
Depression in primary school, % (*n*/*N*)	0.10 (315/319 839)	0.09 (290/307 584)
Depression in primary school as a per cent of all cases of depression, % (*n*/*N*)	3.0 (315/10 458)	1.25 (290/23 040)
Self-harm, % (*n*/*N*)	1.48 (4736/319 839)	3.64 (11 210/307 584)
Average age, mean (s.d.)	17.46 (3.81)	16.42 (2.89)
Self-harm in primary school, % (*n*/*N*)	0.11 (362/319 839)	0.11 (330/307 584)
Self-harm in primary school as a per cent of all cases of self-harm, %	7.6 (362/4746)	3.2 (330/11 210)
*Confounding variables*		
ADHD, % (*n*/*N*)	1.81 (5782/319 839)	0.55 (1689/307 584)
Age, mean (s.d.)	10.00 (3.63)	12.23 (4.75)
ADHD in primary school, % (*n*/*N*)	1.34 (4301/319 839)	0.29 (895/307 584)
ADHD in primary school as a per cent of all cases of ADHD, %	74 (4301/5782)	53 (895/1689)
Conduct disorder	3.55(11 341/319 839)	1.70 (685/307 584)
Average age, mean (s.d.)	9.73 (3.47)	12.41 (4.68)
Stimulants in primary school, % (*n*/*N*)	2.71 (8672/319 839)	0.88 (2719/307 584)
Stimulants in primary school as a per cent of all cases of stimulant use, %	76.5 (8672/11 341)	51.9 (2719/5238)
Intellectual disability, % (*n*/*N*)	0.33 (1042/319 839)	0.18 (541/307 584)
Age, mean (s.d.)	13.77 (5.11)	14.55 (5.08)
Intellectual disability in primary school, % (*n*/*N*)	0.14 (438/319 839)	0.05 (158/307 584)
Intellectual disability in primary school as a per cent of all cases of intellectual disability, %	42 (438/1042)	29 (158/541)
Hypnotic, % (*n*/*N*)	1.19 (3800/31 839)	1.52 (4674/307 584)
Average age, mean (s.d.)	17.58 (5.43)	19.64 (3.94)
Hypnotic in primary school, % (*n*/*N*)	0.24 (756/319 839)	0.09 (271/307 584)
Hypnotic in primary school as a per cent of all cases of hypnotic use, %	19.9 (756/3800)	5.8 (271/4674)
Stimulants, % (*n*/*N*)	1.27 (4051/319 839)	0.28 (850/307 584)
Age, mean (s.d.)	10.67 (3.32)	11.78 (3.98)
Stimulants in primary school, % (*n*/*N*)	0.86 (2747/319 839)	0.16 (490/307 584)
Stimulants in primary school as a per cent of all cases of stimulant use, %	67.8 (2747/4051)	57.6 (490/850)

ADHD, attention-deficit hyperactivity disorder.

**Table 3:** Adjusted hazard ratios for outcomes in adolescence after key stage 2 (KS2)^a^

	Depression after KS2 (age 10–11) Hazard ratio (95% CI) *n* = 626 512	Self-harm after KS2 Hazard ratio (age 10–11) (95% CI) *n* = 626 553
Not achieving at age 7	0.93 (0.87–1.00)	**1.17 (1.07–1.29)**
Not achieving at age 11	**1.26 (1.18–1.34)**	1.03 (0.94–1.12)
Free school meals at age 11	**1.45 (1.36–1.54)**	**1.51 (1.38–1.65)**
Free school meal at age 7	**1.35 (1.27–1.45)**	**1.65 (1.51–1.80)**
Female gender	**2.85 (2.71–2.99)**	**4.38 (4.06–4.78)**
ADHD (in primary school)	1.13 (0.88–1.44)	**1.84 (1.38–2.45)**
Conduct disorder (in primary school)	**1.57 (1.33–1.86)**	**1.71 (1.37–2.14)**
Intellectual difficulties (in primary school)	**0.35 (0.14–0.84)**	0.45 (0.16–1.22)
Hypnotic prescription (primary school)	1.27 (0.82–1.95)	1.07 (0.63–1.79)
Stimulant prescription (in primary school)	0.92 (0.61–1.35)	1.29 (0.83–2.00)

Results in bold are significant.

a. Adjusted for attention-deficit hyperactivity disorder (ADHD), conduct disorder and intellectual difficulties.

**Table 4:** Adjusted hazard ratios for outcomes in adolescence after key stage (KS) 3 and after KS4^a^

	Depression after KS3 (age 14–20) *n* = 624 608	Depression before KS3 (age 12–14) *N* = 620 840	Self-harm after KS3 (age 14–20) *N* = 624 466	Self-harm before KS3 (age 12–14) *N* = 621 151	Depression after KS4 (age 16–20) *n* = 618 936	Depression before KS4 (age 12–16) *N* = 602 038	Self-harm after KS4 (age 16–20) *N* = 619 064	Self-harm before KS4 (age 12–16) *N* = 619 413
Not achieving at age 14	**1.38 (1.28–1.48)**	**1.41 (1.30–1.53)**	**1.61 (1.45–1.79)**	**1.63 (1.43–1.87)**	–	–	–	–
Not achieving at age 16	**–**	**–**	**–**	**–**	**1.50 (1.30–1.73)**	**1.44 (1.04–1.96)**	**1.67 (1.26–2.19)**	**2.11 (1.48–3.03)**

Results in bold are significant.

a. Adjusted for KS1, KS2, free school meals and female gender.

Depression is predominantly occurring in the later teenage years and therefore there is a long time lag between poor education attainment in primary school and diagnosis of depression in later adolescence (Table 5).

**Table 5:** Incidence of depression and self-harm (i.e. age at first diagnosis of depression or self-harm)

Age, years	Depression, *n* (%)	Self-harm, *n* (%)
11	351 (1.06)	255 (1.64)
12	677 (2.05)	313 (0.95)
13	1227 (3.7)	1450 (9.3)
14	2372 (7.2)	1011 (6.51)
15	3300 (10)	2919 (18.8)
16	4139 (12.6)	1590 (10.24)
17	5982 (18.1)	1949 (12.5)
18	7021 (21.8)	1618 (10.4)
19	7206 (21.8)	1357 (8.7)
1–11 or 19–20	618 (1.69)	2792 (20.96)
Total	32 893	15 254

### Self-harm

There were 15 946 (2.4%) individuals with a record of self-harm in adolescence. In addition, there were 692 excluded who had a record of self-harm in primary school, these children were more often boys. The crude hazard ratio (HR) of demonstrating self-harm behaviours (years of follow up 11 467 479) if not achieving KS2 (age 11) was 1.38 (95% CI 1.31–1.43) and the adjusted HR was 1.03 (95% CI 0.94–1.12) (Table 3). However, in adolescence, girls were four times more likely to self-harm than boys (HR=4.38, 95% CI 4.06–4.78). Young people who self-harmed in adolescence were achieving as well at age 10/11 (KS2) as those who do not self-harm in adolescence (HR=1.03 (0.94–1.12) (Table 3). However, they were not achieving as well at age 7 (HR=1.17, 95% CI 1.07–1.29) compared with those who do not self-harm. This suggests (moving from less likely to achieve to equally likely compared with those who do not self-harm) that they were improving and progressing well in primary school. However, a decline is evident during secondary school, where they are 60% less likely to achieve their KS3 and KS4 before there is any record of self-harm (compared with those who do not self-harm). Self-harm in adolescence was also associated with being female and a diagnosis of conduct disorder in primary school and/or ADHD. Young people who self-harm decline in educational attainment before they are diagnosed with self-harm behaviors (HR=1.61, 95% CI 1.45–1.79) (Table 4). The results show there is a close temporal proximity in the decline in academic attainment and associated self-harm behaviour (Table 5).

## Discussion

### Main findings

This study found that the association between educational attainment and depression may differ from the association seen with self-harm. The young people who went on to receive a diagnosis of depression/symptoms of depression were already declining in educational attainment at school for a number of years before the diagnosis was made. The peak period of incidence for depression in this study was 18–19 years but a decline in academic attainment was apparent from primary school and through secondary school (Table 5).

However, among those who self-harm there was no evidence of a decline in educational attainment in primary school. In fact, the children who self-harmed in the teenage years were improving in primary school, being less likely to achieve at age 7 (HR=1.17) but just as able at age 11 as those who do not self-harm. The young people who self-harm started declining in education attainment in secondary school almost concurrently with their self-harming behaviour, for example the peak time of self-harm behaviour was aged 15 (Table 5) and this is also the time when these children were not achieving in school (Table 4). This suggests a number of possibilities: that those who self-harm may be well supported in primary school but lose this support in secondary school or perhaps find the transition to secondary school a challenge or that self-harm is associated with a more acute contemporaneous problem occurring in later teenage years (i.e. that academic decline could be a ‘symptom’ of another problem in those who self-harm).

### Comparison with findings from other studies

A survey of 12- to 15-year-olds in school in Australia and the USA found self-harming behaviour to be particularly related to late or completed puberty, girls and self-cutting.^25^ The association of self-harm with puberty may be related to a recognised neurodevelopmental stage in adolescents^26^ with structural and functional changes associated with increased risk of emotional disorders, risky behaviours and vulnerability to peer pressure that may go some way to explaining the timing of the decline in attainment in those who self-harm compared with those with depression. Various models exist to explain the psychological processes that underlie self-harming behaviours (stress-diathesis; interpersonal model of suicide; motivational–volitional model)^27^ and suggest that the degree to which people feel defeated is associated with self-harming behaviours and this may imply a bi-directional mechanism between attainment and self-harm. However, both depression and self-harming are associated with deprivation and are more common in girls and those with conduct disorder/ADHD.

### Depression in primary schools

The study results suggest a temporal association between academic attainment and subsequent depression in children and young people. In addition, our study data raises the possibility that depression symptoms and low mood are being missed in primary school, possibly because the adults around them are not recognising their depressive symptoms. Indeed, epidemiological cohort studies illustrate that most depression goes unrecognised and untreated in young people despite the fact that rapid early specialist treatment ameliorates later outcomes.^17^^,^^28^ Potentially, academic decline or disengagement acts as an early symptom of depression and this may then predict subsequent full-blown episodes – subthreshold depressive symptoms predict later major depressive episodes.^29^^,^^30^ Another possibility is that academic decline may co-occur with other depression risk factors (such as poverty, stressful life events, family adversity) and thereby increase the likelihood of subsequent depression. Indeed, evidence and theory suggest that social and familial risk factors for depression tend to co-occur^31^^–^^33^ and the effects of exposure to multiple risk factors may be cumulative.^34^ Self-harm is also associated with adverse family circumstances (abuse, neglect, poor attachment) and such factors are known to have an impact on educational attainment but this would not explain the difference in timing for depression and self-harm.

### Implications

This study has important implications suggesting declining academic attainment may be an indicator that interventions aimed at emotional and social development could improve and potentially reduce the development of future mental health problems. Depression occurs through multiple pathways and one route is the ‘failure’ route whereby social and academic problems affect an individual's self-perceptions and increase vulnerability to depression.^35^ It is recognised that depression is a complex multifactorial disorder. This implies different routes to depression for different individuals and indeed, we note that in 6307 (30%) out of 20 759 individuals with depression this was associated with non-achievement of key stage 2 meaning that 70% of those with diagnosed depression did achieve key stage 2. Therefore, academic decline is only one of a number of factors associated with depression. Nonetheless, our longitudinal results suggest that it may be a useful factor to be incorporated into the development of risk profiling/prediction tools in the future. It is plausible that helping children improve their academic attainment and supporting them at an early stage may help protect against future depression.

### Strengths and limitations

This study is novel in bringing together education data and health data on a national level to give a large sample that can look at the temporal relationship between academic attainment and depression, and provides a longitudinal cohort of real-world observational data. However, it must be recognised that data was not collected originally for research and this means diagnosis and data collection is not consistent in all periods of time and across all areas. Differences in coding with time and with GP practice will affect prevalence levels of outcomes and confounders with time. Healthcare and education standards change with time, and these changes are not reflected or accounted for in these results. For example, recent changes in mental health awareness and provision of school counsellors in the UK and other interventions will mean children in secondary school in 2014 will have a different experience from those in secondary school in the mid-1990s. This is an observational study and as such the findings would need to be repeated in other populations to confirm validity and repeatability. Importantly, this study did not examine undiagnosed depression or self-harm and so the findings are only relevant to those who present at services with depression or self-harm. This raises an obvious limitation in terms of predicting future events using only diagnosed disease when it is known that the majority of depression in childhood/adolescence is untreated and undiagnosed. Therefore, the strength of the association of decline in educational attainment at school and depression may be underestimated with this study.

In addition, this study does not attempt to look at the severity or length of time a person has depression or have been self-harming. We did not examine how severe the depression is that is associated with educational decline. We did not impute missing data for educational attainment as the available data did not give good estimates of attainment because the data known to be good predictors of child attainment, such as maternal education and mobility, were unavailable. The data-set we were using was large, comprising over 800 000 children and adolescents and it was therefore assumed that the majority of data was missing at random (for example living in England at time of exam) and so we have made the assumption that excluding those with missing education data will not bias the findings. The results of the current study reflect presentation to primary care, recognition by GPs and the way in which depression and self-harm in children and young people is recorded in primary care. However, this is likely to be an underestimation since routine data does not capture individuals with whom depression or self-harm is discussed, but not recorded. This is common feature of all routinely collected database studies.

Finally, children born in the early 1990s will not have their key stage 1 (age 7) and key stage 2 (age 11) records included in the educational data-set, which only started in 2005. Their data will not be included in analysis looking at key stage 1 and 2 but their data will be included in analysis looking at a later key stage where data is captured. Therefore, the later key stage analysis will contain a different mix of children compared with the earlier key stage analysis.

In summary, impaired academic performance precedes overt clinical symptoms of depression. The findings from this study show that this decline may be seen as early as primary school and imply that preventing these children becoming disengaged from the educational system may be critical in modifying the development of mental disorder and, perhaps economic inactivity.^36^ The findings from this study suggest either (a) children with depression are not detected in the primary school years and this affects subsequent academic performance, or (b) that prolonged academic decline in the early school years is a risk factor for depression in adolescence or arguably that it is possible that (c) another trait, such as family factors is associated with both the development of depression and academic achievement. In contrast, there was no evidence that academic decline in primary school was associated with future self-harm behaviour although declining attainment in secondary school is associated with self-harm behaviour.

## Supplementary material

For supplementary material accompanying this paper visit https://doi.org/10.1192/bjp.2017.69. click here to view supplementary material
