# Supplementary material for: School achievement as a predictor of depression and self-harm in adolescence: linked education and health record study
Source: Br J Psychiatry. 2018 Mar 6;212(4):215–21. doi: 10.1192/bjp.2017.69 (PMC7557863; doi:10.1192/bjp.2017.69)
Supplement: Supplementary file 1 [file S0007125017000691sup.zip › S0007125017000691sup001.docx]

**Supplementary file 1: Read codes/ICD10 codes**

| **GP: DEPRESSION** |
| --- |
| \| E112. \| Single major depressive episode \| \| --- \| --- \| \| E1120 \| Single major depressive episode, unspecified \| \| E1121 \| Single major depressive episode, mild \| \| E1122 \| Single major depressive episode, moderate \| \| E1123 \| Single major depressive episode, severe, without psychosis \| \| E1125 \| Single major depressive episode, partial or unspec remission \| \| E1126 \| Single major depressive episode, in full remission \| \| E112z \| Single major depressive episode NOS \| \| E113. \| Recurrent major depressive episode \| \| E1130 \| Recurrent major depressive episodes, unspecified \| \| E1131 \| Recurrent major depressive episodes, mild \| \| E1132 \| Recurrent major depressive episodes, moderate \| \| E1133 \| Recurrent major depressive episodes, severe, no psychosis \| \| E1135 \| Recurrent major depressive episodes,partial/unspec remission \| \| E1136 \| Recurrent major depressive episodes, in full remission \| \| E1137 \| Recurrent depression \| \| E113z \| Recurrent major depressive episode NOS \| \| E118. \| Seasonal affective disorder \| \| E135. \| Agitated depression \| \| E2003 \| Anxiety with depression \| \| E204. \| Neurotic depression reactive type \| \| E291. \| Prolonged depressive reaction \| \| E2B.. \| Depressive disorder NEC \| \| E2B0. \| Postviral depression \| \| E2B1. \| Chronic depression \| \| Eu32. \| [X]Depressive episode \| \| Eu320 \| [X]Mild depressive episode \| \| Eu321 \| [X]Moderate depressive episode \| \| Eu322 \| [X]Severe depressive episode without psychotic symptoms \| \| Eu324 \| [X]Mild depression \| \| Eu32y \| [X]Other depressive episodes \| \| Eu32z \| [X]Depressive episode, unspecified \| \| Eu33. \| [X]Recurrent depressive disorder \| \| Eu330 \| [X]Recurrent depressive disorder, current episode mild \| \| Eu331 \| [X]Recurrent depressive disorder, current episode moderate \| \| Eu332 \| [X]Recurr depress disorder cur epi severe without psyc sympt \| \| Eu334 \| [X]Recurrent depressive disorder, currently in remission \| \| Eu33y \| [X]Other recurrent depressive disorders \| \| **depression symptom** \| \| \| 1B17. \| Depressed \| \| 1B1U. \| Symptoms of depression \| \| 1BQ.. \| Loss of capacity for enjoyment \| \| 1BT.. \| Depressed mood \| \| 1BU.. \| Loss of hope for the future \| \| 2257 \| O/E - depressed \| \|  \|  \| |
| **GP: SELF-HARM** |
| \| SL... \| Poisoning \| \| --- \| --- \| \| TK601 \| Self inflicted lacerations to wrist \| \| TN... \| Injury undetermined whether accidentally/purposely inflicted \| \| TN0.. \| Injury ?accidental, poisoning by solid/liquid substances \| \| TN00. \| Injury ?accidental, poisoning by analgesic or anti-pyretic \| \| TN01. \| Injury ?accidental, poisoning by barbiturate \| \| TN010 \| Injury ?accidental poisoning by Amylobarbitone \| \| TN011 \| Injury ?accidental poisoning by Barbitone \| \| TN012 \| Injury ?accidental poisoning by Butabarbitone \| \| TN013 \| Injury ?accidental poisoning by Pentobarbitone \| \| TN014 \| Injury ? accidental poisoning by Phenobarbitone \| \| TN015 \| Injury ? accidental poisoning by Quinalbarbitone \| \| TN01z \| Injury ?accidental poisoning by barbiturate NOS \| \| TN02. \| Injury ?accidental, poisoning by other sedative/hypnotic \| \| TN03. \| Injury ?accidental, poisoning by tranquilliser/psychotropic \| \| TN04. \| Injury ?accidental, poisoning by other spec drug/medicament \| \| TN05. \| Injury ?accidental, poisoning by drug or medicament NOS \| \| TN06. \| Injury ?accidental, poisoning by corrosive/caustic substance \| \| TN07. \| Injury ?accidental, poisoning by agricultural chemicals \| \| TN08. \| Injury ?accidental, poisoning by arsenic or its compounds \| \| TN0z. \| Injury ?accidental, poisoning by solid or liquid subst NOS \| \| TN1.. \| Injury ?accidental, poisoning by gases in domestic use \| \| TN10. \| Injury ?accidental, poisoning by gas distributed by pipeline \| \| TN11. \| Injury ?accidental, poisoning by liquid petrol gas \| \| TN1y. \| Injury ?accidental, poisoning by other utility gas \| \| TN1z. \| Injury ?accidental, poisoning by gas in domestic use NOS \| \| TN2.. \| Injury ?accidental, poisoning by other gases \| \| TN20. \| Injury ?accidental, poisoning by motor vehicle exhaust gas \| \| TN21. \| Injury ?accidental, poisoning by other carbon monoxide \| \| TN2y. \| Injury ?accidental, poisoning by other spec gas or vapour \| \| TN2z. \| Injury ?accidental, poisoning by gas or vapour NOS \| \| TN3.. \| Injury ?accidental, hanging, strangulation and suffocation \| \| TN30. \| Injury ?accidental, hanging \| \| TN31. \| Injury ?accidental, suffocation by plastic bag \| \| TN3y. \| Injury ?accidental, other means of hang/strangle/suffocate \| \| TN3z. \| Injury ?accidental, hanging/strangulation/suffocation NOS \| \| TN4.. \| Injury ?accidental, drowning \| \| TN5.. \| Injury ?accidental, by firearms and explosives \| \| TN50. \| Injury ?accidental, by handgun \| \| TN51. \| Injury ?accidental, by shotgun \| \| TN52. \| Injury ?accidental, by hunting rifle \| \| TN53. \| Injury ?accidental, by military firearms \| \| TN54. \| Injury ?accidental, by other firearm \| \| TN55. \| Injury ?accidental, by explosive \| \| TN5z. \| Injury ?accidental, by firearm or explosive NOS \| \| TN6.. \| Injury ?accidental, by cutting and stabbing instruments \| \| TN60. \| Injury ?accidental, by cutting instrument \| \| TN61. \| Injury ?accidental, by stabbing instrument \| \| TN6z. \| Injury ?accidental, by cutting or stabbing instrument NOS \| \| TN7.. \| Injury ?accidental, fall from high place \| \| TN70. \| Injury ?accidental, fall from residential premises \| \| TN71. \| Injury ?accidental, fall from other man-made structure \| \| TN72. \| Injury ?accidental, fall from natural site \| \| TN7z. \| Injury ?accidental, fall from high place NOS \| \| TN8.. \| Injury ?accidental, by other means \| \| TN80. \| Injury ?accidental, by jumping or lying before moving object \| \| TN800 \| Injury ?accidental, by jumping before moving object \| \| TN801 \| Injury ?accidental, by lying before moving object \| \| TN80z \| Injury ?accidental, jumping/lying before moving object NOS \| \| TN81. \| Injury ?accidental, by burns or fire \| \| TN82. \| Injury ?accidental, by scald \| \| TN83. \| Injury ?accidental, by extremes of cold \| \| TN84. \| Injury ?accidental, by electrocution \| \| TN85. \| Injury ?accidental, by crashing of motor vehicle \| \| TN86. \| Injury ?accidental, by crashing of aircraft \| \| TN87. \| Injury ?accidental, by caustic substances, except poisoning \| \| TN8y. \| Injury ?accidental, by other specified means \| \| TN8z. \| Injury ?accidental, by means NOS \| \| TN9.. \| Injury ?accidental, late effects \| \| TNz.. \| Injury undetermined accidental or purposely inflicted NOS \| \| U2... \| [X]Intentional self-harm \| \| U20.. \| [X]Intentional self poisoning/exposure to noxious substances \| \| U200. \| [X]Intent self poison/exposure to nonopioid analgesic \| \| U2000 \| [X]Int self poison/exposure to nonopioid analgesic at home \| \| U2001 \| [X]Intent self poison nonopioid analgesic at res institut \| \| U2002 \| [X]Int self poison nonopioid analges school/pub admin area \| \| U2003 \| [X]Int self poison nonopioid analges in sport/athletic area \| \| U2004 \| [X]Intent self pois nonopioid analgesic in street/highway \| \| U2005 \| [X]Intent self pois nonopioid analgesic trade/service area \| \| U2006 \| [X]Int self pois nonopioid analgesic indust/construct area \| \| U2007 \| [X]Int self poison/exposure to nonopioid analgesic on farm \| \| U200y \| [X]Int self poison nonopioid analgesic other spec place \| \| U200z \| [X]Intent self poison nonopioid analgesic unspecif place \| \| U201. \| [X]Intent self poison/exposure to antiepileptic \| \| U2010 \| [X]Int self poison/exposure to antiepileptic at home \| \| U2011 \| [X]Intent self poison antiepileptic at res institut \| \| U2012 \| [X]Intent self pois nonopioid analges school/pub admin area \| \| U2013 \| [X]Int self poison antiepileptic in sport/athletic area \| \| U2014 \| [X]Intent self pois antiepileptic in street/highway \| \| U2015 \| [X]Intent self pois antiepileptic trade/service area \| \| U2016 \| [X]Int self poison antiepileptic indust/construct area \| \| U2017 \| [X]Int self poison/exposure to antiepileptic on farm \| \| U201y \| [X]Intent self poison antiepileptic other spec place \| \| U201z \| [X]Intent self poison antiepileptic unspecif place \| \| U202. \| [X]Intent self poison/exposure to sedative hypnotic \| \| U2020 \| [X]Int self poison/exposure to sedative hypnotic at home \| \| U2021 \| [X]Intent self poison sedative hypnotic at res institut \| \| U2022 \| [X]Int self poison sedative hypnotic school/pub admin area \| \| U2023 \| [X]Int self poison sedative hypnotic in sport/athletic area \| \| U2024 \| [X]Intent self pois sedative hypnotic in street/highway \| \| U2025 \| [X]Intent self pois sedative hypnotic trade/service area \| \| U2026 \| [X]Int self pois sedative hypnotic indust/construct area \| \| U2027 \| [X]Int self poison/exposure to sedative hypnotic on farm \| \| U202y \| [X]Int self poison sedative hypnotic other spec place \| \| U202z \| [X]Intent self poison sedative hypnotic unspecif place \| \| U203. \| [X]Intent self poison/exposure to antiparkinson drug \| \| U2030 \| [X]Int self poison/exposure to antiparkinson drug at home \| \| U2031 \| [X]Intent self poison antiparkinson drug at res institut \| \| U2032 \| [X]Int self poison antparkinson drug school/pub admin area \| \| U2033 \| [X]Int self poison antparkinson drug in sport/athletic area \| \| U2034 \| [X]Intent self pois antiparkinson drug in street/highway \| \| U2035 \| [X]Intent self pois antiparkinson drug trade/service area \| \| U2036 \| [X]Int self pois antiparkinson drug indust/construct area \| \| U2037 \| [X]Int self poison/exposure to antiparkinson drug on farm \| \| U203y \| [X]Int self poison antiparkinson drug other spec place \| \| U203z \| [X]Intent self poison antiparkinson drug unspecif place \| \| U204. \| [X]Intent self poison/exposure to psychotropic drug \| \| U2040 \| [X]Int self poison/exposure to psychotropic drug at home \| \| U2041 \| [X]Intent self poison psychotropic drug at res institut \| \| U2042 \| [X]Int self poison psychotropic drug school/pub admin area \| \| U2043 \| [X]Int self poison psychotropic drug in sport/athletic area \| \| U2044 \| [X]Intent self pois psychotropic drug in street/highway \| \| U2045 \| [X]Intent self pois psychotropic drug trade/service area \| \| U2046 \| [X]Int self pois psychotropic drug indust/construct area \| \| U2047 \| [X]Int self poison/exposure to psychotropic drug on farm \| \| U204y \| [X]Int self poison psychotropic drug other spec place \| \| U204z \| [X]Intent self poison psychotropic drug unspecif place \| \| U205. \| [X]Intent self poison/exposure to narcotic drug \| \| U2050 \| [X]Int self poison/exposure to narcotic drug at home \| \| U2051 \| [X]Intent self poison narcotic drug at res institut \| \| U2052 \| [X]Int self poison narcotic drug school/pub admin area \| \| U2053 \| [X]Int self poison narcotic drug in sport/athletic area \| \| U2054 \| [X]Intent self pois narcotic drug in street/highway \| \| U2055 \| [X]Intent self pois narcotic drug trade/service area \| \| U2056 \| [X]Int self pois narcotic drug indust/construct area \| \| U2057 \| [X]Int self poison/exposure to narcotic drug on farm \| \| U205y \| [X]Int self poison narcotic drug other spec place \| \| U205z \| [X]Intent self poison narcotic drug unspecif place \| \| U206. \| [X]Intent self poison/exposure to hallucinogen \| \| U2060 \| [X]Int self poison/exposure to hallucinogen at home \| \| U2061 \| [X]Intent self poison hallucinogen at res institut \| \| U2062 \| [X]Int self poison hallucinogenschool/pub admin area \| \| U2063 \| [X]Int self poison hallucinogenin sport/athletic area \| \| U2064 \| [X]Intent self pois hallucinogen in street/highway \| \| U2065 \| [X]Intent self pois hallucinogen trade/service area \| \| U2066 \| [X]Int self pois hallucinogen indust/construct area \| \| U2067 \| [X]Int self poison/exposure to hallucinogen on farm \| \| U206y \| [X]Int self poison hallucinogen other spec place \| \| U206z \| [X]Intent self poison hallucinogen unspecif place \| \| U207. \| [X]Intent self poison/exposure to oth autonomic drug \| \| U2070 \| [X]Int self poison/exposure to oth autonomic drug at home \| \| U2071 \| [X]Intent self poison oth autonomic drug at res institut \| \| U2072 \| [X]Int self poison oth autonom drug school/pub admin area \| \| U2073 \| [X]Int self poison oth autonom drug in sport/athletic area \| \| U2074 \| [X]Intent self pois oth autonomic drug in street/highway \| \| U2075 \| [X]Intent self pois oth autonomic drug trade/service area \| \| U2076 \| [X]Int self pois oth autonomic drug indust/construct area \| \| U2077 \| [X]Int self poison/exposure to oth autonomic drug on farm \| \| U207y \| [X]Int self poison oth autonomic drug other spec place \| \| U207z \| [X]Intent self poison oth autonomic drug unspecif place \| \| U208. \| [X]Int self poison/exposure to other/unspec drug/medicament \| \| U2080 \| [X]Int self poison/exposure to oth/unsp drug/medicam home \| \| U2081 \| [X]Intent self poison oth/unsp drug/medicam res institut \| \| U2082 \| [X]Int self poison oth/uns drug/med school/pub admin area \| \| U2083 \| [X]Int self poison oth/uns drug/med in sport/athletic area \| \| U2084 \| [X]Intent self pois oth/unsp drug/medic in street/highway \| \| U2085 \| [X]Intent self pois oth/unsp drug/medic trade/service area \| \| U2086 \| [X]Int self pois oth/unsp drug/medic indust/construct area \| \| U2087 \| [X]Int self poison/exposure to oth/unsp drug/medic on farm \| \| U208y \| [X]Int self poison oth/unsp drug/medic other spec place \| \| U208z \| [X]Intent self poison oth/unsp drug/medic unspecif place \| \| U20A. \| [X]Intentional self poison organ solvent,halogen hydrocarb \| \| U20A0 \| [X]Intent self pois organ solvent,halogen hydrocarb, home \| \| U20A1 \| [X]Int self poison org solvent,halogen hydrocarb,res instit \| \| U20A2 \| [X]Int self poison org solvent,halogen hydrocarb, school \| \| U20A4 \| [X]Int self poison org solvent,halogen hydrocarb,in highway \| \| U20A5 \| [X]Int self poison org solvent,halogen hydrocarb,trade area \| \| U20A6 \| [X]Int self pois org solvent,halogen hydrocarb,indust area \| \| U20A7 \| [X]Int self poison org solvent,halogen hydrocarb,on farm \| \| U20Ay \| [X]Int self pois org solv,halogen hydrocarb,oth spec place \| \| U20Az \| [X]Int self pois org solv,halogen hydrocarb, unspec place \| \| U20B. \| [X]Intent self poison/exposure to other gas/vapour \| \| U20B0 \| [X]Int self poison/exposure to other gas/vapour at home \| \| U20B1 \| [X]Intent self poison other gas/vapour at res institut \| \| U20B2 \| [X]Int self poison other gas/vapour school/pub admin area \| \| U20B3 \| [X]Int self poison other gas/vapour in sport/athletic area \| \| U20B4 \| [X]Intent self pois other gas/vapour in street/highway \| \| U20B5 \| [X]Intent self pois other gas/vapour trade/service area \| \| U20B6 \| [X]Int self pois other gas/vapour indust/construct area \| \| U20B7 \| [X]Int self poison/exposure to other gas/vapour on farm \| \| U20By \| [X]Int self poison other gas/vapour other spec place \| \| U20Bz \| [X]Intent self poison other gas/vapour unspecif place \| \| U20C. \| [X]Intent self poison/exposure to pesticide \| \| U20C0 \| [X]Int self poison/exposure to pesticide at home \| \| U20C1 \| [X]Intent self poison pesticide at res institut \| \| U20C2 \| [X]Int self poison pesticide school/pub admin area \| \| U20C3 \| [X]Int self poison pesticide in sport/athletic area \| \| U20C4 \| [X]Intent self pois pesticide in street/highway \| \| U20C5 \| [X]Intent self pois pesticide trade/service area \| \| U20C6 \| [X]Int self pois pesticide indust/construct area \| \| U20C7 \| [X]Int self poison/exposure to pesticide on farm \| \| U20Cy \| [X]Int self poison pesticide other spec place \| \| U20Cz \| [X]Intent self poison pesticide unspecif place \| \| U20y. \| [X]Intent self poison/exposure to unspecif chemical \| \| U20y0 \| [X]Int self poison/exposure to unspecif chemical at home \| \| U20y1 \| [X]Intent self poison unspecif chemical at res institut \| \| U20y2 \| [X]Int self poison unspecif chemical school/pub admin area \| \| U20y3 \| [X]Int self poison unspecif chemical in sport/athletic area \| \| U20y4 \| [X]Intent self pois unspecif chemical in street/highway \| \| U20y5 \| [X]Intent self pois unspecif chemical trade/service area \| \| U20y6 \| [X]Int self pois unspecif chemical indust/construct area \| \| U20y7 \| [X]Int self poison/exposure to unspecif chemical on farm \| \| U20yy \| [X]Int self poison unspecif chemical other spec place \| \| U20yz \| [X]Intent self poison unspecif chemical unspecif place \| \| U21.. \| [X]Intent self harm by hanging strangulation / suffocation \| \| U210. \| [X]Intent self harm by hanging strangulat/suffocat occ home \| \| U211. \| [X]Intent self harm by hangng strangult/suffoct resid instit \| \| U212. \| [X]Inten slf harm hang strang/suffc sch oth ins/pub adm area \| \| U213. \| [X]Intent self harm by hang strangl/suffc sport/athlet area \| \| U214. \| [X]Intent self harm by hangng strangult/suffoct street/h'way \| \| U215. \| [X]Intent self harm by hang strangl/suffc trade/service area \| \| U216. \| [X]Intent self harm by hang strangl/suffc indust/constr area \| \| U217. \| [X]Intent self harm by hanging strangulat/suffocat occ farm \| \| U21y. \| [X]Intent self harm by hangng strangul/suffoct oth spec plce \| \| U21z. \| [X]Intent self harm by hangng strangul/suffoct unspecif plce \| \| U22.. \| [X]Intentional self harm by drowning and submersion \| \| U220. \| [X]Intent self harm by drowning/submersion occurrn at home \| \| U221. \| [X]Intent self harm by drowning/submersn occ resid instit'n \| \| U222. \| [X]Intent self harm drown/submers occ sch/ins/pub adm area \| \| U223. \| [X]Intent self harm by drown/submersn occ sport/athlet area \| \| U224. \| [X]Intent self harm by drowning/submersn occ street/highway \| \| U225. \| [X]Intent self harm by drown/submersn occ trade/servce area \| \| U226. \| [X]Intent self harm by drown/submers occ indust/constr area \| \| U227. \| [X]Intent self harm by drowning/submersion occurrn on farm \| \| U22y. \| [X]Intent self harm by drown/submersn occ oth specif place \| \| U22z. \| [X]Intent self harm by drown/submersn occ unspecified place \| \| U23.. \| [X]Intentional self harm by handgun discharge \| \| U230. \| [X]Intention self harm by handgun discharge occurrn at home \| \| U231. \| [X]Intent self harm by handgun disch occ in resid instit'n \| \| U232. \| [X]Intent self harm h'gun disch occ sch oth ins/pub adm area \| \| U233. \| [X]Intent self harm by handgun disch occ sport/athlet area \| \| U234. \| [X]Intent self harm by handgun disch occ on street/highway \| \| U235. \| [X]Intent self harm by handgun disch occ trade/service area \| \| U236. \| [X]Intent self harm by handgun disch occ indust/constr area \| \| U237. \| [X]Intention self harm by handgun discharge occurrn on farm \| \| U23y. \| [X]Intent self harm by handgun disch occ at oth specif plce \| \| U23z. \| [X]Intent self harm by handgun disch occ at unspecif place \| \| U24.. \| [X]Intent self harm by rifle shotgun/larger firearm disch \| \| U240. \| [X]Intent self harm rifle sh'gun/largr firarm disch occ home \| \| U241. \| [X]Int self harm rifl s'gun/lrg frarm disch occ resid instit \| \| U242. \| [X]Int slf hrm rifl s'gun/lrg frarm dis sch/ins/pub adm area \| \| U243. \| [X]Int self harm rifl s'gun/lrg frarm disch sprt/athlet area \| \| U244. \| [X]Int self harm rifl s'gun/lrg frarm disch occ street/h'way \| \| U245. \| [X]Int self harm rifl s'gun/lrg frarm disch trad/servce area \| \| U246. \| [X]Int slf hrm rifl s'gun/lrg frarm disch indust/constr area \| \| U247. \| [X]Intent self harm rifle sh'gun/largr firarm disch occ farm \| \| U24y. \| [X]Int self harm rifl s'gun/lrg frarm disch oth specif place \| \| U24z. \| [X]Int self harm rifl s'gun/lrg frarm disch occ unspec place \| \| U25.. \| [X]Intent self harm by other/unspecified firearm discharge \| \| U250. \| [X]Intent self harm oth/unspecif firearm disch occ at home \| \| U251. \| [X]Intent self harm oth/unsp firearm disch occ resid instit \| \| U252. \| [X]Inten self harm oth/uns firarm disch sch/ins/pub adm area \| \| U253. \| [X]Inten self harm oth/uns firearm disch occ sprt/athl area \| \| U254. \| [X]Intent self harm oth/unsp firearm disch occ street/h'way \| \| U255. \| [X]Intent self harm oth/uns firearm disch trade/servce area \| \| U256. \| [X]Inten self harm oth/uns firearm disch indust/constr area \| \| U257. \| [X]Intent self harm oth/unspecif firearm disch occ on farm \| \| U25y. \| [X]Intent self harm oth/unsp firearm disch oth specif place \| \| U25z. \| [X]Intent self harm oth/unsp firearm disch occ unspecif plce \| \| U26.. \| [X]Intentional self harm by explosive material \| \| U260. \| [X]Intention self harm by explosive material occurrn home \| \| U261. \| [X]Intention self harm by explosiv materl occ resid instit \| \| U262. \| [X]Intent self harm by explosiv materl sch/ins/pub adm area \| \| U263. \| [X]Intent self harm by explosv materl occ sport/athlet area \| \| U264. \| [X]Intention self harm by explosiv materl occ street/highway \| \| U265. \| [X]Intent self harm by explosv materl occ trade/servce area \| \| U266. \| [X]Intent self harm by explosv materl occ indust/constr area \| \| U267. \| [X]Intention self harm by explosive material occurrn farm \| \| U26y. \| [X]Intent self harm by explosiv materl occ oth specif place \| \| U26z. \| [X]Intent self harm by explosiv materl occ unspecif place \| \| U27.. \| [X]Intentional self harm by smoke, fire and flames \| \| U270. \| [X]Intention self harm by smoke fire/flames occurrn at home \| \| U271. \| [X]Intent self harm by smoke fire/flame occ resid instit'n \| \| U272. \| [X]Intent self harm by smoke fire/flame sch/ins/pub adm area \| \| U273. \| [X]Intent self harm by smok fire/flam occ sport/athlet area \| \| U274. \| [X]Intent self harm by smoke fire/flame occ street/highway \| \| U275. \| [X]Intent self harm by smok fire/flam occ trade/servce area \| \| U276. \| [X]Intent self harm by smok fire/flam occ indust/constr area \| \| U277. \| [X]Intention self harm by smoke fire/flames occurrn on farm \| \| U27y. \| [X]Intent self harm by smoke fire/flame occ oth specif plce \| \| U27z. \| [X]Intent self harm by smoke fire/flames occ unspecif place \| \| U28.. \| [X]Intentional self harm by steam hot vapours / hot objects \| \| U280. \| [X]Intent self harm by steam hot vapour/hot obj occ at home \| \| U281. \| [X]Intent self harm by steam hot vapour/obj occ resid instit \| \| U282. \| [X]Int self harm by steam hot vapor/obj sch/ins/pub adm area \| \| U283. \| [X]Int self harm by steam hot vapour/obj occ sport/athl area \| \| U284. \| [X]Intent self harm by steam hot vapour/obj occ street/h'way \| \| U285. \| [X]Int self harm by steam hot vapour/obj trade/service area \| \| U286. \| [X]Int self harm by steam hot vapour/obj indust/constr area \| \| U287. \| [X]Intent self harm by steam hot vapour/hot obj occ on farm \| \| U28y. \| [X]Intent self harm by steam hot vapour/obj oth specif place \| \| U28z. \| [X]Intent self harm by steam hot vapour/obj occ unspec place \| \| U29.. \| [X]Intentional self harm by sharp object \| \| U290. \| [X]Intentional self harm by sharp object occurrence at home \| \| U291. \| [X]Intent self harm by sharp object occ resident instit'n \| \| U292. \| [X]Intent self harm sharp obj occ sch oth ins/pub adm area \| \| U293. \| [X]Intent self harm by sharp object occ sports/athlet area \| \| U294. \| [X]Intention self harm by sharp object occ street/highway \| \| U295. \| [X]Intent self harm by sharp object occ trade/service area \| \| U296. \| [X]Intent self harm by sharp object occ indust/constr area \| \| U297. \| [X]Intentional self harm by sharp object occurrence on farm \| \| U29y. \| [X]Intention self harm by sharp object occ oth specif place \| \| U29z. \| [X]Intentional self harm by sharp object occ unspecif place \| \| U2A.. \| [X]Intentional self harm by blunt object \| \| U2A0. \| [X]Intentional self harm by blunt object occurrence at home \| \| U2A1. \| [X]Intent self harm by blunt object occ resident instit'n \| \| U2A2. \| [X]Intent self harm blunt obj occ sch oth ins/pub adm area \| \| U2A3. \| [X]Intent self harm by blunt object occ sports/athlet area \| \| U2A4. \| [X]Intention self harm by blunt object occ street/highway \| \| U2A5. \| [X]Intent self harm by blunt object occ trade/service area \| \| U2A6. \| [X]Intent self harm by blunt object occ indust/constr area \| \| U2A7. \| [X]Intentional self harm by blunt object occurrence on farm \| \| U2Ay. \| [X]Intention self harm by blunt object occ oth specif place \| \| U2Az. \| [X]Intentional self harm by blunt object occ unspecif place \| \| U2B.. \| [X]Intentional self harm by jumping from a high place \| \| U2B0. \| [X]Intent self harm by jumping from high place occ at home \| \| U2B1. \| [X]Intent self harm by jump from high place occ resid instit \| \| U2B2. \| [X]Int self harm jump fr high place sch oth ins/pub adm area \| \| U2B3. \| [X]Intent self harm by jump from high place sport/athl area \| \| U2B4. \| [X]Intent self harm by jump from high place occ street/h'way \| \| U2B5. \| [X]Intent self harm by jump from high place trad/servce area \| \| U2B6. \| [X]Int self harm by jump from high place indust/constr area \| \| U2B7. \| [X]Intent self harm by jumping from high place occ on farm \| \| U2By. \| [X]Int self harm by jump from high place occ oth specif plce \| \| U2Bz. \| [X]Int self harm by jump from high place occ unspecif place \| \| U2C.. \| [X]Intent self harm by jumping / lying before moving object \| \| U2C0. \| [X]Intent self harm by jump/lying befor moving obj occ home \| \| U2C1. \| [X]Int self harm jump/lying befr mov obje occ resid instit'n \| \| U2C2. \| [X]Int self harm jump/lying bef mov obj sch/ins/pub adm area \| \| U2C3. \| [X]Int self harm jump/lying bef mov obj occ sprt/athlet area \| \| U2C4. \| [X]Int self harm jump/lying befr mov obje occ street/highway \| \| U2C5. \| [X]Int self harm jump/lying bef mov obj occ trad/servce area \| \| U2C6. \| [X]Int self harm jump/lying befr mov obj indust/constr area \| \| U2C7. \| [X]Intent self harm by jump/lying befor moving obj occ farm \| \| U2Cy. \| [X]Int self harm jump/lying bef mov obje occ oth specif plce \| \| U2Cz. \| [X]Int self harm jump/lying bef mov obje occ unspecif place \| \| U2D.. \| [X]Intentional self harm by crashing of motor vehicle \| \| U2D0. \| [X]Intent self harm by crash of motor vehicl occurrn at home \| \| U2D1. \| [X]Intent self harm by crash motor vehicl occ resid instit'n \| \| U2D2. \| [X]Int self harm crash motor vehicl occ sch/ins/pub adm area \| \| U2D3. \| [X]Intent self harm by crash motor vehicl occ sprt/athl area \| \| U2D4. \| [X]Intent self harm by crash motor vehicl occ street/highway \| \| U2D5. \| [X]Intent self harm crash motor vehicl occ trade/servce area \| \| U2D6. \| [X]Intent self harm crash motor vehic occ indust/constr area \| \| U2D7. \| [X]Intent self harm by crash of motor vehicl occurrn on farm \| \| U2Dy. \| [X]Intent self harm by crash motor vehic occ oth specif plce \| \| U2Dz. \| [X]Intent self harm by crash motor vehic occ unspecif place \| \| U2E.. \| [X]Self mutilation \| \| U2y.. \| [X]Intentional self harm by other specified means \| \| U2y0. \| [X]Intentionl self harm by oth specif means occurrn at home \| \| U2y1. \| [X]Intent self harm by oth specif means occ resid instit'n \| \| U2y2. \| [X]Intent self harm oth specif mean occ sch/ins/pub adm area \| \| U2y3. \| [X]Intent self harm by oth specif means occ sport/athl area \| \| U2y4. \| [X]Intent self harm by oth specif means occ street/highway \| \| U2y5. \| [X]Intent self harm by oth specif means occ trad/servce area \| \| U2y6. \| [X]Intent self harm oth specif means occ indust/constr area \| \| U2y7. \| [X]Intentionl self harm by oth specif means occurrn on farm \| \| U2yy. \| [X]Intent self harm oth specif means occ oth specif place \| \| U2yz. \| [X]Intent self harm by oth specif means occ unspecif place \| \| U2z.. \| [X]Intentional self harm by unspecified means \| \| U2z0. \| [X]Intentional self harm by unspecif means occurrn at home \| \| U2z1. \| [X]Intent self harm by unspecif means occurrn resid instit'n \| \| U2z2. \| [X]Intent self harm by unspec mean occ sch/ins/pub adm area \| \| U2z3. \| [X]Intent self harm unspecif means occurrn sport/athlet area \| \| U2z4. \| [X]Intent self harm by unspecif means occurrn street/highway \| \| U2z5. \| [X]Intent self harm unspecif means occurrn trade/servce area \| \| U2z6. \| [X]Intent self harm unspecif mean occurrn indust/constr area \| \| U2z7. \| [X]Intentional self harm by unspecif means occurrn on farm \| \| U2zy. \| [X]Intent self harm by unspecif means occ oth specif place \| \| U2zz. \| [X]Intent self harm by unspecif means occ at unspecif place \| \| U4... \| [X]Event of undetermined intent \| \| U40.. \| [X]Poisoning/expos to noxious substance,undetermined intent \| \| U400. \| [X]Poisoning/exposure, ? intent, to nonopioid analgesic \| \| U4000 \| [X]Poison/exposure ?intent, to nonopioid analgesic at home \| \| U4001 \| [X]Pois/expos ?intent to nonopioid analgesic at res institut \| \| U4002 \| [X]Pois/exp ?intent nonopioid analges school/pub admin area \| \| U4003 \| [X]Pois/exp ?intent nonopioid analges in sport/athletic area \| \| U4004 \| [X]Pois/expos ?intent nonopioid analgesic in street/highway \| \| U4005 \| [X]Pois/expos ?intent nonopioid analgesic trade/service area \| \| U4006 \| [X]Pois/exp ?intent nonopioid analges indust/construct area \| \| U4007 \| [X]Poison/exposure ?intent, to nonopioid analgesic on farm \| \| U400y \| [X]Pois/exp ?intent to nonopioid analgesic other spec place \| \| U400z \| [X]Pois/expos ?intent to nonopioid analgesic unspecif place \| \| U401. \| [X]Poisoning/exposure, ? intent, to antiepileptic \| \| U4010 \| [X]Poison/exposure ?intent, to antiepileptic at home \| \| U4011 \| [X]Pois/expos ?intent to antiepileptic at res institut \| \| U4012 \| [X]Pois/expos ?intent nonopioid analgs school/pub admin area \| \| U4013 \| [X]Pois/exp ?intent antiepileptics in sport/athletic area \| \| U4014 \| [X]Pois/expos ?intent antiepileptic in street/highway \| \| U4015 \| [X]Pois/expos ?intent antiepileptic trade/service area \| \| U4016 \| [X]Pois/exp ?intent antiepileptic indust/construct area \| \| U4017 \| [X]Poison/exposure ?intent, to antiepileptic on farm \| \| U401y \| [X]Pois/expos ?intent to antiepileptic other spec place \| \| U401z \| [X]Pois/expos ?intent to antiepileptic unspecif place \| \| U402. \| [X]Poisoning/exposure, ? intent, to sedative hypnotic \| \| U4020 \| [X]Poison/exposure ?intent, to sedative hypnotic at home \| \| U4021 \| [X]Pois/expos ?intent to sedative hypnotic at res institut \| \| U4022 \| [X]Pois/exp ?intent sedative hypnotic school/pub admin area \| \| U4023 \| [X]Pois/exp ?intent sedative hypnotic in sport/athletic area \| \| U4024 \| [X]Pois/expos ?intent sedative hypnotic in street/highway \| \| U4025 \| [X]Pois/expos ?intent sedative hypnotic trade/service area \| \| U4026 \| [X]Pois/exp ?intent sedative hypnotic indust/construct area \| \| U4027 \| [X]Poison/exposure ?intent, to sedative hypnotic on farm \| \| U402y \| [X]Pois/exp ?intent to sedative hypnotic other spec place \| \| U402z \| [X]Pois/expos ?intent to sedative hypnotic unspecif place \| \| U403. \| [X]Poisoning/exposure, ? intent, to antiparkinson drug \| \| U4030 \| [X]Poison/exposure ?intent, to antiparkinson drug at home \| \| U4031 \| [X]Pois/expos ?intent to antiparkinson drug at res institut \| \| U4032 \| [X]Pois/exp ?intent antparkinson drug school/pub admin area \| \| U4033 \| [X]Pois/exp ?intent antparkinson drug in sport/athletic area \| \| U4034 \| [X]Pois/expos ?intent antiparkinson drug in street/highway \| \| U4035 \| [X]Pois/expos ?intent antiparkinson drug trade/service area \| \| U4036 \| [X]Pois/exp ?intent antiparkinson drug indust/construct area \| \| U4037 \| [X]Poison/exposure ?intent, to antiparkinson drug on farm \| \| U403y \| [X]Pois/exp ?intent to antiparkinson drug other spec place \| \| U403z \| [X]Pois/expos ?intent to antiparkinson drug unspecif place \| \| U404. \| [X]Poisoning/exposure, ? intent, to psychotropic drug \| \| U4040 \| [X]Poison/exposure ?intent, to psychotropic drug at home \| \| U4041 \| [X]Pois/expos ?intent to psychotropic drug at res institut \| \| U4042 \| [X]Pois/exp ?intent psychotropic drug school/pub admin area \| \| U4043 \| [X]Pois/exp ?intent psychotropic drug in sport/athletic area \| \| U4044 \| [X]Pois/expos ?intent psychotropic drug in street/highway \| \| U4045 \| [X]Pois/expos ?intent psychotropic drug trade/service area \| \| U4046 \| [X]Pois/exp ?intent psychotropic drug indust/construct area \| \| U4047 \| [X]Poison/exposure ?intent, to psychotropic drug on farm \| \| U404y \| [X]Pois/exp ?intent to psychotropic drug other spec place \| \| U404z \| [X]Pois/expos ?intent to psychotropic drug unspecif place \| \| U405. \| [X]Poisoning/exposure, ? intent, to narcotic drug \| \| U4050 \| [X]Poison/exposure ?intent, to narcotic drug at home \| \| U4051 \| [X]Pois/expos ?intent to narcotic drug at res institut \| \| U4052 \| [X]Pois/exp ?intent narcotic drug school/pub admin area \| \| U4053 \| [X]Pois/exp ?intent narcotic drug in sport/athletic area \| \| U4054 \| [X]Pois/expos ?intent narcotic drug in street/highway \| \| U4055 \| [X]Pois/expos ?intent narcotic drug trade/service area \| \| U4056 \| [X]Pois/expos ?intent narcotic drug indust/construct area \| \| U4057 \| [X]Poison/exposure ?intent, to narcotic drug on farm \| \| U405y \| [X]Pois/exp ?intent to narcotic drug other spec place \| \| U405z \| [X]Pois/expos ?intent to narcotic drug unspecif place \| \| U406. \| [X]Poisoning/exposure, ? intent, to hallucinogen \| \| U4060 \| [X]Poison/exposure ?intent, to hallucinogen at home \| \| U4061 \| [X]Pois/expos ?intent to hallucinogen at res institut \| \| U4062 \| [X]Pois/exp ?intent hallucinogenschool/pub admin area \| \| U4063 \| [X]Pois/exp ?intent hallucinogenin sport/athletic area \| \| U4064 \| [X]Pois/expos ?intent hallucinogen in street/highway \| \| U4065 \| [X]Pois/expos ?intent hallucinogen trade/service area \| \| U4066 \| [X]Poison/expos, ?intent, hallucinogen indust/construct area \| \| U4067 \| [X]Poison/exposure ?intent, to hallucinogen on farm \| \| U406y \| [X]Pois/exp ?intent to hallucinogen other spec place \| \| U406z \| [X]Pois/expos ?intent to hallucinogen unspecif place \| \| U407. \| [X]Poisoning/exposure, ? intent, to other autonomic drug \| \| U4070 \| [X]Poison/exposure ?intent, to oth autonomic drug at home \| \| U4071 \| [X]Pois/expos ?intent to oth autonomic drug at res institut \| \| U4072 \| [X]Pois/exp ?intent oth autonom drug school/pub admin area \| \| U4073 \| [X]Pois/exp ?intent oth autonom drug in sport/athletic area \| \| U4074 \| [X]Pois/expos ?intent oth autonomic drug in street/highway \| \| U4075 \| [X]Pois/expos ?intent oth autonomic drug trade/service area \| \| U4076 \| [X]Pois/exp ?intent oth autonomic drug indust/construct area \| \| U4077 \| [X]Poison/exposure ?intent, to oth autonomic drug on farm \| \| U407y \| [X]Pois/exp ?intent to oth autonomic drug other spec place \| \| U407z \| [X]Pois/expos ?intent to oth autonomic drug unspecif place \| \| U408. \| [X]Poison/exposure, ?intent, to other/unspec drug/medicament \| \| U4080 \| [X]Poison/exposure ?intent, to oth/unsp drug/medicam home \| \| U4081 \| [X]Pois/expos ?intent to oth/unsp drug/medicam res institut \| \| U4082 \| [X]Pois/exp ?intent oth/uns drug/med school/pub admin area \| \| U4083 \| [X]Pois/exp ?intent oth/uns drug/med in sport/athletic area \| \| U4084 \| [X]Pois/expos ?intent oth/unsp drug/medic in street/highway \| \| U4085 \| [X]Pois/expos ?intent oth/unsp drug/medic trade/service area \| \| U4086 \| [X]Pois/exp ?intent oth/unsp drug/medic indust/construc area \| \| U4087 \| [X]Poison/exposure ?intent, to oth/unsp drug/medic on farm \| \| U408y \| [X]Pois/exp ?intent to oth/unsp drug/medic other spec place \| \| U408z \| [X]Pois/expos ?intent to oth/unsp drug/medic unspecif place \| \| U40A. \| [X]Pois/exposure,?intent,to organ solvent,halogen hydrocarb \| \| U40A0 \| [X]Pois/expos ?intent organ solvent,halogen hydrocarb, home \| \| U40A1 \| [X]Pois/exp ?intent org solvent,halogen hydrocarb,res instit \| \| U40A2 \| [X]Pois/exp ?intent org solvent,halogen hydrocarb, school \| \| U40A3 \| [X]Pois/exp ?intent org solvent,halogen hydrocarb,sport area \| \| U40A4 \| [X]Pois/exp ?intent org solvent,halogen hydrocarb,in highway \| \| U40A5 \| [X]Pois/exp ?intent org solvent,halogen hydrocarb,trade area \| \| U40A6 \| [X]Pois/exp ?intent org solv,halogen hydrocarb,indust area \| \| U40A7 \| [X]Pois/exp ?intent org solvent,halogen hydrocarb,on farm \| \| U40Ay \| [X]Pois/exp ?intent org solv,halogen hydrocarb,oth sp place \| \| U40Az \| [X]Pois/exp ?intent org solv,halogen hydrocarb, unspec place \| \| U40B. \| [X]Poisoning/exposure, ? intent, to other gas/vapour \| \| U40B0 \| [X]Poison/exposure ?intent, to other gas/vapour at home \| \| U40B1 \| [X]Pois/expos ?intent to other gas/vapour at res institut \| \| U40B2 \| [X]Pois/exp ?intent other gas/vapour school/pub admin area \| \| U40B3 \| [X]Pois/exp ?intent other gas/vapour in sport/athletic area \| \| U40B4 \| [X]Pois/expos ?intent other gas/vapour in street/highway \| \| U40B5 \| [X]Pois/expos ?intent other gas/vapour trade/service area \| \| U40B6 \| [X]Pois/exp ?intent other gas/vapour indust/construct area \| \| U40B7 \| [X]Poison/exposure ?intent, to other gas/vapour on farm \| \| U40By \| [X]Pois/exp ?intent to other gas/vapour other spec place \| \| U40Bz \| [X]Pois/expos ?intent to other gas/vapour unspecif place \| \| U40C. \| [X]Poisoning/exposure, ? intent, to pesticide \| \| U40C0 \| [X]Poison/exposure ?intent, to pesticide at home \| \| U40C1 \| [X]Pois/expos ?intent to pesticide at res institut \| \| U40C2 \| [X]Pois/exp ?intent pesticide school/pub admin area \| \| U40C3 \| [X]Pois/exp ?intent pesticide in sport/athletic area \| \| U40C4 \| [X]Pois/expos ?intent pesticide in street/highway \| \| U40C5 \| [X]Pois/expos ?intent pesticide trade/service area \| \| U40C6 \| [X]Poison/exposure, ?intent, pesticide indust/construct area \| \| U40C7 \| [X]Poison/exposure ?intent, to pesticide on farm \| \| U40Cy \| [X]Pois/exp ?intent to pesticide other spec place \| \| U40Cz \| [X]Pois/expos ?intent to pesticide unspecif place \| \| U40y. \| [X]Poisoning/exposure, ? intent, to unspecif chemical \| \| U40y0 \| [X]Poison/exposure ?intent, to unspecif chemical at home \| \| U40y1 \| [X]Pois/expos ?intent to unspecif chemical at res institut \| \| U40y2 \| [X]Pois/exp ?intent unspecif chemical school/pub admin area \| \| U40y3 \| [X]Pois/exp ?intent unspecif chemical in sport/athletic area \| \| U40y4 \| [X]Pois/expos ?intent unspecif chemical in street/highway \| \| U40y5 \| [X]Pois/expos ?intent unspecif chemical trade/service area \| \| U40y6 \| [X]Poison/expos ?intent unspec chemic indust/construct area \| \| U40y7 \| [X]Poison/exposure ?intent, to unspecif chemical on farm \| \| U40yy \| [X]Pois/exp ?intent to unspecif chemical other spec place \| \| U40yz \| [X]Pois/expos ?intent to unspecif chemical unspecif place \| \| U41.. \| [X]Hanging strangulation + suffocation undetermined intent \| \| U410. \| [X]Hanging strangulat+suffocat undet intent occurrn at home \| \| U411. \| [X]Hangng strangulat+suffocat undet intent resident instit'n \| \| U412. \| [X]Hang strangl+suffoc undet intent sch oth ins/pub adm area \| \| U413. \| [X]Hangng strangulat+suffocat undet intent sport/athlet area \| \| U414. \| [X]Hangng strangult+suffocat undet intent occ street/highway \| \| U415. \| [X]Hangng strangulat+suffocat undet intent trade/servce area \| \| U416. \| [X]Hangng strangult+suffocat undet intent indust/constr area \| \| U417. \| [X]Hanging strangulat+suffocat undet intent occurrn on farm \| \| U41y. \| [X]Hangng strangult+suffoct undet intent occ oth specif plce \| \| U41z. \| [X]Hangng strangult+suffoct undet intent occ unspecif place \| \| U42.. \| [X]Drowning and submersion, undetermined intent \| \| U420. \| [X]Drowning+submersion undetermined intent occurrn at home \| \| U421. \| [X]Drowning+submersion undetermin intent occ resid instit'n \| \| U422. \| [X]Drown+submersn undeterm intent sch oth ins/pub adm area \| \| U423. \| [X]Drowning+submersion undetermin intent sport/athlet area \| \| U424. \| [X]Drowning+submersion undetermin intent occ street/highway \| \| U425. \| [X]Drowning+submersion undetermin intent trade/service area \| \| U426. \| [X]Drowning+submersion undeterm intent indust/constr area \| \| U427. \| [X]Drowning+submersion undetermined intent occurrn on farm \| \| U42y. \| [X]Drownng+submersion undetermin intent occ oth specif plce \| \| U42z. \| [X]Drownng+submersion undetermin intent occ unspecif place \| \| U43.. \| [X]Handgun discharge, undetermined intent \| \| U430. \| [X]Handgun discharge undetermined intent occurrence at home \| \| U431. \| [X]Handgun disch undeterm intent occurrn resident instit'n \| \| U432. \| [X]Handgun disch undet intent occ sch oth ins/pub adm area \| \| U433. \| [X]Handgun disch undeterm intent occurrn sport/athlet area \| \| U434. \| [X]Handgun disch undeterm intent occurrn on street/highway \| \| U435. \| [X]Handgun disch undeterm intent occurrn trade/service area \| \| U436. \| [X]Handgun disch undeterm intent occurrn indust/constr area \| \| U437. \| [X]Handgun discharge undetermined intent occurrence on farm \| \| U43y. \| [X]Handgun disch undeterm intent occurrn at oth specif plce \| \| U43z. \| [X]Handgun disch undeterm intent occurrn at unspecif plce \| \| U44.. \| [X]Rifle shotgun+larger firearm discharge undetermin intent \| \| U440. \| [X]Rifle shotgun+larger firearm disch undet intent occ home \| \| U441. \| [X]Rifle shotgun+larg firarm disch undet intent resid instit \| \| U442. \| [X]Rifl s'gun+lrg frarm disch undt intn sch/ins/pub adm area \| \| U443. \| [X]Rifl s'gun+lrg firarm disch undet intent sprt/athlet area \| \| U444. \| [X]Rifle shotgun+larg firarm disch undet intent street/h'way \| \| U445. \| [X]Rifl s'gun+lrg firarm disch undet intent trad/servce area \| \| U446. \| [X]Rifl s'gun+lrg frarm disch undet intnt indust/constr area \| \| U447. \| [X]Rifle shotgun+larger firearm disch undet intent occ farm \| \| U44y. \| [X]Rifle s'gun+lrg firarm disch undet intent oth specif plce \| \| U44z. \| [X]Rifle s'gun+lrg firarm disch undet intent unspecif place \| \| U45.. \| [X]Other+unspecified firearm discharge undetermined intent \| \| U450. \| [X]Other+unspecif firearm disch undeterm intent occ at home \| \| U451. \| [X]Oth+unsp firearm disch undeterm intent occ resid instit'n \| \| U452. \| [X]Oth+uns firarm disch undeterm intent sch/ins/pub adm area \| \| U453. \| [X]Oth+unsp firearm disch undeterm intent occ sprt/athl area \| \| U454. \| [X]Oth+unsp firearm disch undeterm intent occ street/highway \| \| U455. \| [X]Oth+uns firarm disch undeterm intent occ trad/servce area \| \| U456. \| [X]Oth+uns firearm disch undeterm intent industr/constr area \| \| U457. \| [X]Other+unspecif firearm disch undeterm intent occ on farm \| \| U45y. \| [X]Oth+unsp firarm disch undeterm intent occ oth specif plce \| \| U45z. \| [X]Oth+unsp firearm disch undeterm intent occ unspecif place \| \| U46.. \| [X]Contact with explosive material, undetermined intent \| \| U460. \| [X]Contact with explosiv materl undeterm intent occ at home \| \| U461. \| [X]Contct with explosiv materl undet intent occ resid instit \| \| U462. \| [X]Contc wth explosv materl undet intnt sch/ins/pub adm area \| \| U463. \| [X]Contct wth explosiv materl undet intnt occ sprt/athl area \| \| U464. \| [X]Contct with explosiv materl undet intent occ street/h'way \| \| U465. \| [X]Contct wth explosiv materl undet intnt trade/service area \| \| U466. \| [X]Contct wth explosiv materl undet intnt indust/constr area \| \| U467. \| [X]Contact with explosiv materl undeterm intent occ on farm \| \| U46y. \| [X]Contct with explosiv materl undet intent oth specif place \| \| U46z. \| [X]Contct wth explosiv materl undet intent occ unspecif plce \| \| U47.. \| [X]Exposure to smoke, fire and flames, undetermined intent \| \| U470. \| [X]Exposure to smoke fire+flame undeterm intent occ at home \| \| U471. \| [X]Exposr to smoke fire+flam undet intent occ resid instit'n \| \| U472. \| [X]Expos to smoke fir+flam undet intent sch/ins/pub adm area \| \| U473. \| [X]Exposr to smok fir+flam undet intent occ sprt/athlet area \| \| U474. \| [X]Exposr to smoke fire+flam undet intent occ street/highway \| \| U475. \| [X]Exposr to smok fir+flam undet intent occ trad/servce area \| \| U476. \| [X]Exposr to smok fir+flam undet intent industr/constr area \| \| U477. \| [X]Exposure to smoke fire+flame undeterm intent occ on farm \| \| U47y. \| [X]Exposr to smoke fir+flam undet intent occ oth specif plce \| \| U47z. \| [X]Exposr to smoke fir+flam undet intent occ unspecif place \| \| U48.. \| [X]Contact with steam hot vapours+objects undetermn intent \| \| U480. \| [X]Contact with steam hot vapour+obj undet intent occ home \| \| U481. \| [X]Contact with steam hot vapour+obj undet intent resid inst \| \| U482. \| [X]Contc wth steam hot vap+obj und intn sch/ins/pub adm area \| \| U483. \| [X]Contc with steam hot vap+obj undet intent sport/athl area \| \| U484. \| [X]Contact with steam hot vap+obj undet intent street/h'way \| \| U485. \| [X]Contc with steam hot vap+obj undet intent trade/serv area \| \| U486. \| [X]Contc wth steam hot vap+obj undet intnt indust/cnstr area \| \| U487. \| [X]Contact with steam hot vapour+obj undet intent occ farm \| \| U48y. \| [X]Contact with steam hot vap+obj undet intent oth spec plce \| \| U48z. \| [X]Contact with steam hot vap+obj undet intent unspecif plce \| \| U49.. \| [X]Contact with sharp object, undetermined intent \| \| U490. \| [X]Contact wth sharp object undetermined intent occ at home \| \| U491. \| [X]Contct wth sharp obj undeterm intent occ residn instit'n \| \| U492. \| [X]Contct wth shrp obj undet intent occ sch/ins/pub adm area \| \| U493. \| [X]Contct wth sharp obj undet intent occ sport/athletic area \| \| U494. \| [X]Contact with sharp obj undeterm intent occ street/highway \| \| U495. \| [X]Contct wth sharp obj undet intent occ trade/service area \| \| U496. \| [X]Contct wth sharp obj undet intent occ industr/constr area \| \| U497. \| [X]Contact wth sharp object undetermined intent occ on farm \| \| U49y. \| [X]Contact with sharp obj undeter intent occ oth specif plce \| \| U49z. \| [X]Contact with sharp obj undeterm intent occ unspecif place \| \| U4A.. \| [X]Contact with blunt object, undetermined intent \| \| U4A0. \| [X]Contact wth blunt object undetermined intent occ at home \| \| U4A1. \| [X]Contct wth blunt obj undeterm intent occ residn instit'n \| \| U4A2. \| [X]Contct wth blnt obj undet intent occ sch/ins/pub adm area \| \| U4A3. \| [X]Contct wth blunt obj undet intent occ sport/athletic area \| \| U4A4. \| [X]Contact with blunt obj undeterm intent occ street/highway \| \| U4A5. \| [X]Contct wth blunt obj undet intent occ trade/service area \| \| U4A6. \| [X]Contct wth blunt obj undet intent occ industr/constr area \| \| U4A7. \| [X]Contact wth blunt object undetermined intent occ on farm \| \| U4Ay. \| [X]Contact with blunt obj undeter intent occ oth specif plce \| \| U4Az. \| [X]Contact with blunt obj undeterm intent occ unspecif place \| \| U4B.. \| [X]Falling jumping/pushed from high place undeterm intent \| \| U4B0. \| [X]Fallng jumpng/push frm high place undet intent occ home \| \| U4B1. \| [X]Fall jump/push frm high place undet intent occ resid inst \| \| U4B2. \| [X]Fall jump/push high plce undet intnt sch/ins/pub adm area \| \| U4B3. \| [X]Fall jump/push frm high plce undt intnt sport/athlet area \| \| U4B4. \| [X]Fall jump/push frm high place undt intnt occ street/h'way \| \| U4B5. \| [X]Fall jump/push frm high plce undt intnt trade/servce area \| \| U4B6. \| [X]Fall jump/push frm high plce undt intn indust/constr area \| \| U4B7. \| [X]Fallng jumpng/push frm high place undet intent occ farm \| \| U4By. \| [X]Fall jump/push frm high plce undt intnt occ oth spec plce \| \| U4Bz. \| [X]Fall jump/push frm high plce undt intnt occ unspecif plce \| \| U4C.. \| [X]Falling lying running befor/into moving obj undet intent \| \| U4C0. \| [X]Fallng lyng run befr/into mov obj undet intent occ home \| \| U4C1. \| [X]Fall ly run befr/into mov obj undet intent resid instit'n \| \| U4C2. \| [X]Fall ly run bef/into mov obj und int sch/ins/pub adm area \| \| U4C3. \| [X]Fall ly run befr/into mov obj undet intent sprt/athl area \| \| U4C4. \| [X]Fall ly run befr/into mov obj undet intent street/highway \| \| U4C5. \| [X]Fall ly run befr/into mov obj undet intent trad/serv area \| \| U4C6. \| [X]Fall ly run befr/into mov obj undet intent industr area \| \| U4C7. \| [X]Fallng lyng run befr/into mov obj undet intent occ farm \| \| U4Cy. \| [X]Fall ly run befr/into mov obj undet intent oth spec place \| \| U4Cz. \| [X]Fall ly run befr/into mov obj undet intent unspecif place \| \| U4D.. \| [X]Crashing of motor vehicle, undetermined intent \| \| U4D0. \| [X]Crashng of motor vehicle undetermined intent occ at home \| \| U4D1. \| [X]Crashng of motor vehicle undeterm intent resident instit \| \| U4D2. \| [X]Crash of motor vehicl undeter intent sch/ins/pub adm area \| \| U4D3. \| [X]Crash of motor vehicle undeterm intent sport/athlet area \| \| U4D4. \| [X]Crashng of motor vehicle undeterm intent street/highway \| \| U4D5. \| [X]Crash of motor vehicle undeterm intent trade/servce area \| \| U4D6. \| [X]Crash of motor vehicle undeterm intent indust/constr area \| \| U4D7. \| [X]Crashng of motor vehicle undetermined intent occ on farm \| \| U4Dy. \| [X]Crashng of motor vehicle undeterm intent oth specif plce \| \| U4Dz. \| [X]Crashng of motor vehicle undeterm intent unspecif place \| \| U4y.. \| [X]Other specified events, undetermined intent \| \| U4y0. \| [X]Other specified event undetermind intent occurrn at home \| \| U4y1. \| [X]Oth specif event undetermin intent occ resident instit'n \| \| U4y2. \| [X]Oth specif event undeter intent occ sch/ins/pub adm area \| \| U4y3. \| [X]Oth specif event undetermin intent occ sport/athlet area \| \| U4y4. \| [X]Oth specif event undetermin intent occ on street/highway \| \| U4y5. \| [X]Oth specif event undetermin intent occ trade/servce area \| \| U4y6. \| [X]Oth specif event undetermin intent occ indust/constr area \| \| U4y7. \| [X]Other specified event undetermind intent occurrn on farm \| \| U4yy. \| [X]Oth specif event undetermin intent occ oth specif place \| \| U4yz. \| [X]Oth specif event undetermin intent occ at unspecif place \| \| U4z.. \| [X]Unspecified event, undetermined intent \| \| U4z0. \| [X]Unspecified event undetermined intent occurrence at home \| \| U4z1. \| [X]Unspecif event undeterm intent occurrn resident instit'n \| \| U4z2. \| [X]Unspec event undeterm intent occ sch oth ins/pub adm area \| \| U4z3. \| [X]Unspecif event undeterm intent occurrn sport/athlet area \| \| U4z4. \| [X]Unspecif event undeterm intent occurrn on street/highway \| \| U4z5. \| [X]Unspecif event undeterm intent occurrn trade/servce area \| \| U4z6. \| [X]Unspecif event undeterm intent occurrn indust/constr area \| \| U4z7. \| [X]Unspecified event undetermined intent occurrence on farm \| \| U4zy. \| [X]Unspecif event undeterm intent occurrn oth specif place \| \| U4zz. \| [X]Unspecif event undeterm intent occurrn unspecif place \| \| U72.. \| [X]Sequel intentn self-harm assault+event of undeterm intent \| \| U722. \| [X]Sequelae of events of undetermined intent \| |
| **PEDW: DEPRESSION** |
| \| F32 \| Depressive episode \| \| --- \| --- \| \| F320 \| Mild depressive episode \| \| F321 \| Moderate depressive episode \| \| F322 \| Severe depressive episode without psychotic symptoms \| \| F323 \| Severe depressive episode with psychotic symptoms \| \| F328 \| Other depressive episodes \| \| F329 \| Depressive episode, unspecified \| \| F33 \| Recurrent depressive disorder \| \| F330 \| Recurrent depressive disorder, current episode mild \| \| F331 \| Recurrent depressive disorder, current episode moderate \| \| F332 \| Recurrent depressive disorder, current episode severe without psychotic symptoms \| \| F333 \| Recurrent depressive disorder, current episode severe with psychotic symptoms \| \| F334 \| Recurrent depressive disorder, currently in remission \| \| F338 \| Other recurrent depressive disorders \| \| F339 \| Recurrent depressive disorder, unspecified \| |
| **PEDW: SELF HARM** |
| \| X600 \| Intentional self-poisoning by and exposure to nonopioid analgesics, antipyretics and antirheumatics \| \| --- \| --- \| \| X601 \| Intentional self-poisoning by and exposure to nonopioid analgesics, antipyretics and antirheumatics \| \| X602 \| Intentional self-poisoning by and exposure to nonopioid analgesics, antipyretics and antirheumatics \| \| X603 \| Intentional self-poisoning by and exposure to nonopioid analgesics, antipyretics and antirheumatics \| \| X604 \| Intentional self-poisoning by and exposure to nonopioid analgesics, antipyretics and antirheumatics \| \| X605 \| Intentional self-poisoning by and exposure to nonopioid analgesics, antipyretics and antirheumatics \| \| X606 \| Intentional self-poisoning by and exposure to nonopioid analgesics, antipyretics and antirheumatics \| \| X607 \| Intentional self-poisoning by and exposure to nonopioid analgesics, antipyretics and antirheumatics \| \| X608 \| Intentional self-poisoning by and exposure to nonopioid analgesics, antipyretics and antirheumatics \| \| X609 \| Intentional self-poisoning by and exposure to nonopioid analgesics, antipyretics and antirheumatics \| \| X61 \| Intentional self-poisoning by and exposure to antiepileptic, sedative-hypnotic, antiparkinsonism and psychotropic drugs, not elsewhere classified \| \| X610 \| Intentional self-poisoning by and exposure to antiepileptic, sedative-hypnotic, antiparkinsonism and psychotropic drugs, not elsewhere classified \| \| X611 \| Intentional self-poisoning by and exposure to antiepileptic, sedative-hypnotic, antiparkinsonism and psychotropic drugs, not elsewhere classified \| \| X612 \| Intentional self-poisoning by and exposure to antiepileptic, sedative-hypnotic, antiparkinsonism and psychotropic drugs, not elsewhere classified \| \| X613 \| Intentional self-poisoning by and exposure to antiepileptic, sedative-hypnotic, antiparkinsonism and psychotropic drugs, not elsewhere classified \| \| X614 \| Intentional self-poisoning by and exposure to antiepileptic, sedative-hypnotic, antiparkinsonism and psychotropic drugs, not elsewhere classified \| \| X615 \| Intentional self-poisoning by and exposure to antiepileptic, sedative-hypnotic, antiparkinsonism and psychotropic drugs, not elsewhere classified \| \| X616 \| Intentional self-poisoning by and exposure to antiepileptic, sedative-hypnotic, antiparkinsonism and psychotropic drugs, not elsewhere classified \| \| X617 \| Intentional self-poisoning by and exposure to antiepileptic, sedative-hypnotic, antiparkinsonism and psychotropic drugs, not elsewhere classified \| \| X618 \| Intentional self-poisoning by and exposure to antiepileptic, sedative-hypnotic, antiparkinsonism and psychotropic drugs, not elsewhere classified \| \| X619 \| Intentional self-poisoning by and exposure to antiepileptic, sedative-hypnotic, antiparkinsonism and psychotropic drugs, not elsewhere classified \| \| X62 \| Intentional self-poisoning by and exposure to narcotics and psychodysleptics [hallucinogens], not elsewhere classified \| \| X620 \| Intentional self-poisoning by and exposure to narcotics and psychodysleptics [hallucinogens], not elsewhere classified \| \| X621 \| Intentional self-poisoning by and exposure to narcotics and psychodysleptics [hallucinogens], not elsewhere classified \| \| X622 \| Intentional self-poisoning by and exposure to narcotics and psychodysleptics [hallucinogens], not elsewhere classified \| \| X623 \| Intentional self-poisoning by and exposure to narcotics and psychodysleptics [hallucinogens], not elsewhere classified \| \| X624 \| Intentional self-poisoning by and exposure to narcotics and psychodysleptics [hallucinogens], not elsewhere classified \| \| X625 \| Intentional self-poisoning by and exposure to narcotics and psychodysleptics [hallucinogens], not elsewhere classified \| \| X626 \| Intentional self-poisoning by and exposure to narcotics and psychodysleptics [hallucinogens], not elsewhere classified \| \| X627 \| Intentional self-poisoning by and exposure to narcotics and psychodysleptics [hallucinogens], not elsewhere classified \| \| X628 \| Intentional self-poisoning by and exposure to narcotics and psychodysleptics [hallucinogens], not elsewhere classified \| \| X629 \| Intentional self-poisoning by and exposure to narcotics and psychodysleptics [hallucinogens], not elsewhere classified \| \| X63 \| Intentional self-poisoning by and exposure to other drugs acting on the autonomic nervous system \| \| X630 \| Intentional self-poisoning by and exposure to other drugs acting on the autonomic nervous system \| \| X631 \| Intentional self-poisoning by and exposure to other drugs acting on the autonomic nervous system \| \| X632 \| Intentional self-poisoning by and exposure to other drugs acting on the autonomic nervous system \| \| X633 \| Intentional self-poisoning by and exposure to other drugs acting on the autonomic nervous system \| \| X634 \| Intentional self-poisoning by and exposure to other drugs acting on the autonomic nervous system \| \| X635 \| Intentional self-poisoning by and exposure to other drugs acting on the autonomic nervous system \| \| X636 \| Intentional self-poisoning by and exposure to other drugs acting on the autonomic nervous system \| \| X637 \| Intentional self-poisoning by and exposure to other drugs acting on the autonomic nervous system \| \| X638 \| Intentional self-poisoning by and exposure to other drugs acting on the autonomic nervous system \| \| X639 \| Intentional self-poisoning by and exposure to other drugs acting on the autonomic nervous system \| \| X64 \| Intentional self-poisoning by and exposure to other and unspecified drugs, medicaments and biological substances \| \| X640 \| Intentional self-poisoning by and exposure to other and unspecified drugs, medicaments and biological substances \| \| X641 \| Intentional self-poisoning by and exposure to other and unspecified drugs, medicaments and biological substances \| \| X642 \| Intentional self-poisoning by and exposure to other and unspecified drugs, medicaments and biological substances \| \| X643 \| Intentional self-poisoning by and exposure to other and unspecified drugs, medicaments and biological substances \| \| X644 \| Intentional self-poisoning by and exposure to other and unspecified drugs, medicaments and biological substances \| \| X645 \| Intentional self-poisoning by and exposure to other and unspecified drugs, medicaments and biological substances \| \| X646 \| Intentional self-poisoning by and exposure to other and unspecified drugs, medicaments and biological substances \| \| X647 \| Intentional self-poisoning by and exposure to other and unspecified drugs, medicaments and biological substances \| \| X648 \| Intentional self-poisoning by and exposure to other and unspecified drugs, medicaments and biological substances \| \| X649 \| Intentional self-poisoning by and exposure to other and unspecified drugs, medicaments and biological substances \| \| X65 \| Intentional self-poisoning by and exposure to alcohol \| \| X650 \| Intentional self-poisoning by and exposure to alcohol \| \| X651 \| Intentional self-poisoning by and exposure to alcohol \| \| X652 \| Intentional self-poisoning by and exposure to alcohol \| \| X653 \| Intentional self-poisoning by and exposure to alcohol \| \| X654 \| Intentional self-poisoning by and exposure to alcohol \| \| X655 \| Intentional self-poisoning by and exposure to alcohol \| \| X656 \| Intentional self-poisoning by and exposure to alcohol \| \| X657 \| Intentional self-poisoning by and exposure to alcohol \| \| X658 \| Intentional self-poisoning by and exposure to alcohol \| \| X659 \| Intentional self-poisoning by and exposure to alcohol \| \| X66 \| Intentional self-poisoning by and exposure to organic solvents and halogenated hydrocarbons and their vapours \| \| X660 \| Intentional self-poisoning by and exposure to organic solvents and halogenated hydrocarbons and their vapours \| \| X661 \| Intentional self-poisoning by and exposure to organic solvents and halogenated hydrocarbons and their vapours \| \| X662 \| Intentional self-poisoning by and exposure to organic solvents and halogenated hydrocarbons and their vapours \| \| X663 \| Intentional self-poisoning by and exposure to organic solvents and halogenated hydrocarbons and their vapours \| \| X664 \| Intentional self-poisoning by and exposure to organic solvents and halogenated hydrocarbons and their vapours \| \| X665 \| Intentional self-poisoning by and exposure to organic solvents and halogenated hydrocarbons and their vapours \| \| X666 \| Intentional self-poisoning by and exposure to organic solvents and halogenated hydrocarbons and their vapours \| \| X667 \| Intentional self-poisoning by and exposure to organic solvents and halogenated hydrocarbons and their vapours \| \| X668 \| Intentional self-poisoning by and exposure to organic solvents and halogenated hydrocarbons and their vapours \| \| X669 \| Intentional self-poisoning by and exposure to organic solvents and halogenated hydrocarbons and their vapours \| \| X67 \| Intentional self-poisoning by and exposure to other gases and vapours \| \| X670 \| Intentional self-poisoning by and exposure to other gases and vapours \| \| X671 \| Intentional self-poisoning by and exposure to other gases and vapours \| \| X672 \| Intentional self-poisoning by and exposure to other gases and vapours \| \| X673 \| Intentional self-poisoning by and exposure to other gases and vapours \| \| X674 \| Intentional self-poisoning by and exposure to other gases and vapours \| \| X675 \| Intentional self-poisoning by and exposure to other gases and vapours \| \| X676 \| Intentional self-poisoning by and exposure to other gases and vapours \| \| X677 \| Intentional self-poisoning by and exposure to other gases and vapours \| \| X678 \| Intentional self-poisoning by and exposure to other gases and vapours \| \| X679 \| Intentional self-poisoning by and exposure to other gases and vapours \| \| X68 \| Intentional self-poisoning by and exposure to pesticides \| \| X680 \| Intentional self-poisoning by and exposure to pesticides \| \| X681 \| Intentional self-poisoning by and exposure to pesticides \| \| X682 \| Intentional self-poisoning by and exposure to pesticides \| \| X683 \| Intentional self-poisoning by and exposure to pesticides \| \| X684 \| Intentional self-poisoning by and exposure to pesticides \| \| X685 \| Intentional self-poisoning by and exposure to pesticides \| \| X686 \| Intentional self-poisoning by and exposure to pesticides \| \| X687 \| Intentional self-poisoning by and exposure to pesticides \| \| X688 \| Intentional self-poisoning by and exposure to pesticides \| \| X689 \| Intentional self-poisoning by and exposure to pesticides \| \| X69 \| Intentional self-poisoning by and exposure to other and unspecified chemicals and noxious substances \| \| X690 \| Intentional self-poisoning by and exposure to other and unspecified chemicals and noxious substances \| \| X691 \| Intentional self-poisoning by and exposure to other and unspecified chemicals and noxious substances \| \| X692 \| Intentional self-poisoning by and exposure to other and unspecified chemicals and noxious substances \| \| X693 \| Intentional self-poisoning by and exposure to other and unspecified chemicals and noxious substances \| \| X694 \| Intentional self-poisoning by and exposure to other and unspecified chemicals and noxious substances \| \| X695 \| Intentional self-poisoning by and exposure to other and unspecified chemicals and noxious substances \| \| X696 \| Intentional self-poisoning by and exposure to other and unspecified chemicals and noxious substances \| \| X697 \| Intentional self-poisoning by and exposure to other and unspecified chemicals and noxious substances \| \| X698 \| Intentional self-poisoning by and exposure to other and unspecified chemicals and noxious substances \| \| X699 \| Intentional self-poisoning by and exposure to other and unspecified chemicals and noxious substances \| \| X70 \| Intentional self-harm by hanging, strangulation and suffocation \| \| X700 \| Intentional self-harm by hanging, strangulation and suffocation \| \| X701 \| Intentional self-harm by hanging, strangulation and suffocation \| \| X702 \| Intentional self-harm by hanging, strangulation and suffocation \| \| X703 \| Intentional self-harm by hanging, strangulation and suffocation \| \| X704 \| Intentional self-harm by hanging, strangulation and suffocation \| \| X705 \| Intentional self-harm by hanging, strangulation and suffocation \| \| X706 \| Intentional self-harm by hanging, strangulation and suffocation \| \| X707 \| Intentional self-harm by hanging, strangulation and suffocation \| \| X708 \| Intentional self-harm by hanging, strangulation and suffocation \| \| X709 \| Intentional self-harm by hanging, strangulation and suffocation \| \| X71 \| Intentional self-harm by drowning and submersion \| \| X710 \| Intentional self-harm by drowning and submersion \| \| X711 \| Intentional self-harm by drowning and submersion \| \| X712 \| Intentional self-harm by drowning and submersion \| \| X713 \| Intentional self-harm by drowning and submersion \| \| X714 \| Intentional self-harm by drowning and submersion \| \| X715 \| Intentional self-harm by drowning and submersion \| \| X716 \| Intentional self-harm by drowning and submersion \| \| X717 \| Intentional self-harm by drowning and submersion \| \| X718 \| Intentional self-harm by drowning and submersion \| \| X719 \| Intentional self-harm by drowning and submersion \| \| X72 \| Intentional self-harm by handgun discharge \| \| X720 \| Intentional self-harm by handgun discharge \| \| X721 \| Intentional self-harm by handgun discharge \| \| X722 \| Intentional self-harm by handgun discharge \| \| X723 \| Intentional self-harm by handgun discharge \| \| X724 \| Intentional self-harm by handgun discharge \| \| X725 \| Intentional self-harm by handgun discharge \| \| X726 \| Intentional self-harm by handgun discharge \| \| X727 \| Intentional self-harm by handgun discharge \| \| X728 \| Intentional self-harm by handgun discharge \| \| X729 \| Intentional self-harm by handgun discharge \| \| X73 \| Intentional self-harm by rifle, shotgun and larger firearm discharge \| \| X730 \| Intentional self-harm by rifle, shotgun and larger firearm discharge \| \| X731 \| Intentional self-harm by rifle, shotgun and larger firearm discharge \| \| X732 \| Intentional self-harm by rifle, shotgun and larger firearm discharge \| \| X733 \| Intentional self-harm by rifle, shotgun and larger firearm discharge \| \| X734 \| Intentional self-harm by rifle, shotgun and larger firearm discharge \| \| X735 \| Intentional self-harm by rifle, shotgun and larger firearm discharge \| \| X736 \| Intentional self-harm by rifle, shotgun and larger firearm discharge \| \| X737 \| Intentional self-harm by rifle, shotgun and larger firearm discharge \| \| X738 \| Intentional self-harm by rifle, shotgun and larger firearm discharge \| \| X739 \| Intentional self-harm by rifle, shotgun and larger firearm discharge \| \| X74 \| Intentional self-harm by other and unspecified firearm discharge \| \| X740 \| Intentional self-harm by other and unspecified firearm discharge \| \| X741 \| Intentional self-harm by other and unspecified firearm discharge \| \| X742 \| Intentional self-harm by other and unspecified firearm discharge \| \| X743 \| Intentional self-harm by other and unspecified firearm discharge \| \| X744 \| Intentional self-harm by other and unspecified firearm discharge \| \| X745 \| Intentional self-harm by other and unspecified firearm discharge \| \| X746 \| Intentional self-harm by other and unspecified firearm discharge \| \| X747 \| Intentional self-harm by other and unspecified firearm discharge \| \| X748 \| Intentional self-harm by other and unspecified firearm discharge \| \| X749 \| Intentional self-harm by other and unspecified firearm discharge \| \| X75 \| Intentional self-harm by explosive material \| \| X750 \| Intentional self-harm by explosive material \| \| X751 \| Intentional self-harm by explosive material \| \| X752 \| Intentional self-harm by explosive material \| \| X753 \| Intentional self-harm by explosive material \| \| X754 \| Intentional self-harm by explosive material \| \| X755 \| Intentional self-harm by explosive material \| \| X756 \| Intentional self-harm by explosive material \| \| X757 \| Intentional self-harm by explosive material \| \| X758 \| Intentional self-harm by explosive material \| \| X759 \| Intentional self-harm by explosive material \| \| X76 \| Intentional self-harm by smoke, fire and flames \| \| X760 \| Intentional self-harm by smoke, fire and flames \| \| X761 \| Intentional self-harm by smoke, fire and flames \| \| X762 \| Intentional self-harm by smoke, fire and flames \| \| X763 \| Intentional self-harm by smoke, fire and flames \| \| X764 \| Intentional self-harm by smoke, fire and flames \| \| X765 \| Intentional self-harm by smoke, fire and flames \| \| X766 \| Intentional self-harm by smoke, fire and flames \| \| X767 \| Intentional self-harm by smoke, fire and flames \| \| X768 \| Intentional self-harm by smoke, fire and flames \| \| X769 \| Intentional self-harm by smoke, fire and flames \| \| X77 \| Intentional self-harm by steam, hot vapours and hot objects \| \| X770 \| Intentional self-harm by steam, hot vapours and hot objects \| \| X771 \| Intentional self-harm by steam, hot vapours and hot objects \| \| X772 \| Intentional self-harm by steam, hot vapours and hot objects \| \| X773 \| Intentional self-harm by steam, hot vapours and hot objects \| \| X774 \| Intentional self-harm by steam, hot vapours and hot objects \| \| X775 \| Intentional self-harm by steam, hot vapours and hot objects \| \| X776 \| Intentional self-harm by steam, hot vapours and hot objects \| \| X777 \| Intentional self-harm by steam, hot vapours and hot objects \| \| X778 \| Intentional self-harm by steam, hot vapours and hot objects \| \| X779 \| Intentional self-harm by steam, hot vapours and hot objects \| \| X78 \| Intentional self-harm by sharp object \| \| X780 \| Intentional self-harm by sharp object \| \| X781 \| Intentional self-harm by sharp object \| \| X782 \| Intentional self-harm by sharp object \| \| X783 \| Intentional self-harm by sharp object \| \| X784 \| Intentional self-harm by sharp object \| \| X785 \| Intentional self-harm by sharp object \| \| X786 \| Intentional self-harm by sharp object \| \| X787 \| Intentional self-harm by sharp object \| \| X788 \| Intentional self-harm by sharp object \| \| X789 \| Intentional self-harm by sharp object \| \| X79 \| Intentional self-harm by blunt object \| \| X790 \| Intentional self-harm by blunt object \| \| X791 \| Intentional self-harm by blunt object \| \| X792 \| Intentional self-harm by blunt object \| \| X793 \| Intentional self-harm by blunt object \| \| X794 \| Intentional self-harm by blunt object \| \| X795 \| Intentional self-harm by blunt object \| \| X796 \| Intentional self-harm by blunt object \| \| X797 \| Intentional self-harm by blunt object \| \| X798 \| Intentional self-harm by blunt object \| \| X799 \| Intentional self-harm by blunt object \| \| X80 \| Intentional self-harm by jumping from a high place \| \| X800 \| Intentional self-harm by jumping from a high place \| \| X801 \| Intentional self-harm by jumping from a high place \| \| X802 \| Intentional self-harm by jumping from a high place \| \| X803 \| Intentional self-harm by jumping from a high place \| \| X804 \| Intentional self-harm by jumping from a high place \| \| X805 \| Intentional self-harm by jumping from a high place \| \| X806 \| Intentional self-harm by jumping from a high place \| \| X807 \| Intentional self-harm by jumping from a high place \| \| X808 \| Intentional self-harm by jumping from a high place \| \| X809 \| Intentional self-harm by jumping from a high place \| \| X81 \| Intentional self-harm by jumping or lying before moving object \| \| X810 \| Intentional self-harm by jumping or lying before moving object \| \| X811 \| Intentional self-harm by jumping or lying before moving object \| \| X812 \| Intentional self-harm by jumping or lying before moving object \| \| X813 \| Intentional self-harm by jumping or lying before moving object \| \| X814 \| Intentional self-harm by jumping or lying before moving object \| \| X815 \| Intentional self-harm by jumping or lying before moving object \| \| X816 \| Intentional self-harm by jumping or lying before moving object \| \| X817 \| Intentional self-harm by jumping or lying before moving object \| \| X818 \| Intentional self-harm by jumping or lying before moving object \| \| X819 \| Intentional self-harm by jumping or lying before moving object \| \| X82 \| Intentional self-harm by crashing of motor vehicle \| \| X820 \| Intentional self-harm by crashing of motor vehicle \| \| X821 \| Intentional self-harm by crashing of motor vehicle \| \| X822 \| Intentional self-harm by crashing of motor vehicle \| \| X823 \| Intentional self-harm by crashing of motor vehicle \| \| X824 \| Intentional self-harm by crashing of motor vehicle \| \| X825 \| Intentional self-harm by crashing of motor vehicle \| \| X826 \| Intentional self-harm by crashing of motor vehicle \| \| X827 \| Intentional self-harm by crashing of motor vehicle \| \| X828 \| Intentional self-harm by crashing of motor vehicle \| \| X829 \| Intentional self-harm by crashing of motor vehicle \| \| X83 \| Intentional self-harm by other specified means \| \| X830 \| Intentional self-harm by other specified means \| \| X831 \| Intentional self-harm by other specified means \| \| X832 \| Intentional self-harm by other specified means \| \| X833 \| Intentional self-harm by other specified means \| \| X834 \| Intentional self-harm by other specified means \| \| X835 \| Intentional self-harm by other specified means \| \| X836 \| Intentional self-harm by other specified means \| \| X837 \| Intentional self-harm by other specified means \| \| X838 \| Intentional self-harm by other specified means \| \| X839 \| Intentional self-harm by other specified means \| \| X84 \| Intentional self-harm by unspecified means \| \| X840 \| Intentional self-harm by unspecified means \| \| X841 \| Intentional self-harm by unspecified means \| \| X842 \| Intentional self-harm by unspecified means \| \| X843 \| Intentional self-harm by unspecified means \| \| X844 \| Intentional self-harm by unspecified means \| \| X845 \| Intentional self-harm by unspecified means \| \| X846 \| Intentional self-harm by unspecified means \| \| X847 \| Intentional self-harm by unspecified means \| \| X848 \| Intentional self-harm by unspecified means \| \| X849 \| Intentional self-harm by unspecified means \| \| Y10 \| Poisoning by and exposure to nonopioid analgesics, antipyretics and antirheumatics, undetermined intent \| \| Y100 \| Poisoning by and exposure to nonopioid analgesics, antipyretics and antirheumatics, undetermined intent \| \| Y101 \| Poisoning by and exposure to nonopioid analgesics, antipyretics and antirheumatics, undetermined intent \| \| Y102 \| Poisoning by and exposure to nonopioid analgesics, antipyretics and antirheumatics, undetermined intent \| \| Y103 \| Poisoning by and exposure to nonopioid analgesics, antipyretics and antirheumatics, undetermined intent \| \| Y104 \| Poisoning by and exposure to nonopioid analgesics, antipyretics and antirheumatics, undetermined intent \| \| Y105 \| Poisoning by and exposure to nonopioid analgesics, antipyretics and antirheumatics, undetermined intent \| \| Y106 \| Poisoning by and exposure to nonopioid analgesics, antipyretics and antirheumatics, undetermined intent \| \| Y107 \| Poisoning by and exposure to nonopioid analgesics, antipyretics and antirheumatics, undetermined intent \| \| Y108 \| Poisoning by and exposure to nonopioid analgesics, antipyretics and antirheumatics, undetermined intent \| \| Y109 \| Poisoning by and exposure to nonopioid analgesics, antipyretics and antirheumatics, undetermined intent \| \| Y11 \| Poisoning by and exposure to antiepileptic, sedative-hypnotic, antiparkinsonism and psychotropic drugs, not elsewhere classified, undetermined intent \| \| Y110 \| Poisoning by and exposure to antiepileptic, sedative-hypnotic, antiparkinsonism and psychotropic drugs, not elsewhere classified, undetermined intent \| \| Y111 \| Poisoning by and exposure to antiepileptic, sedative-hypnotic, antiparkinsonism and psychotropic drugs, not elsewhere classified, undetermined intent \| \| Y112 \| Poisoning by and exposure to antiepileptic, sedative-hypnotic, antiparkinsonism and psychotropic drugs, not elsewhere classified, undetermined intent \| \| Y113 \| Poisoning by and exposure to antiepileptic, sedative-hypnotic, antiparkinsonism and psychotropic drugs, not elsewhere classified, undetermined intent \| \| Y114 \| Poisoning by and exposure to antiepileptic, sedative-hypnotic, antiparkinsonism and psychotropic drugs, not elsewhere classified, undetermined intent \| \| Y115 \| Poisoning by and exposure to antiepileptic, sedative-hypnotic, antiparkinsonism and psychotropic drugs, not elsewhere classified, undetermined intent \| \| Y116 \| Poisoning by and exposure to antiepileptic, sedative-hypnotic, antiparkinsonism and psychotropic drugs, not elsewhere classified, undetermined intent \| \| Y117 \| Poisoning by and exposure to antiepileptic, sedative-hypnotic, antiparkinsonism and psychotropic drugs, not elsewhere classified, undetermined intent \| \| Y118 \| Poisoning by and exposure to antiepileptic, sedative-hypnotic, antiparkinsonism and psychotropic drugs, not elsewhere classified, undetermined intent \| \| Y119 \| Poisoning by and exposure to antiepileptic, sedative-hypnotic, antiparkinsonism and psychotropic drugs, not elsewhere classified, undetermined intent \| \| Y12 \| Poisoning by and exposure to narcotics and psychodysleptics [hallucinogens], not elsewhere classified, undetermined intent \| \| Y120 \| Poisoning by and exposure to narcotics and psychodysleptics [hallucinogens], not elsewhere classified, undetermined intent \| \| Y121 \| Poisoning by and exposure to narcotics and psychodysleptics [hallucinogens], not elsewhere classified, undetermined intent \| \| Y122 \| Poisoning by and exposure to narcotics and psychodysleptics [hallucinogens], not elsewhere classified, undetermined intent \| \| Y123 \| Poisoning by and exposure to narcotics and psychodysleptics [hallucinogens], not elsewhere classified, undetermined intent \| \| Y124 \| Poisoning by and exposure to narcotics and psychodysleptics [hallucinogens], not elsewhere classified, undetermined intent \| \| Y125 \| Poisoning by and exposure to narcotics and psychodysleptics [hallucinogens], not elsewhere classified, undetermined intent \| \| Y126 \| Poisoning by and exposure to narcotics and psychodysleptics [hallucinogens], not elsewhere classified, undetermined intent \| \| Y127 \| Poisoning by and exposure to narcotics and psychodysleptics [hallucinogens], not elsewhere classified, undetermined intent \| \| Y128 \| Poisoning by and exposure to narcotics and psychodysleptics [hallucinogens], not elsewhere classified, undetermined intent \| \| Y129 \| Poisoning by and exposure to narcotics and psychodysleptics [hallucinogens], not elsewhere classified, undetermined intent \| \| Y13 \| Poisoning by and exposure to other drugs acting on the autonomic nervous system, undetermined intent \| \| Y130 \| Poisoning by and exposure to other drugs acting on the autonomic nervous system, undetermined intent \| \| Y131 \| Poisoning by and exposure to other drugs acting on the autonomic nervous system, undetermined intent \| \| Y132 \| Poisoning by and exposure to other drugs acting on the autonomic nervous system, undetermined intent \| \| Y133 \| Poisoning by and exposure to other drugs acting on the autonomic nervous system, undetermined intent \| \| Y134 \| Poisoning by and exposure to other drugs acting on the autonomic nervous system, undetermined intent \| \| Y135 \| Poisoning by and exposure to other drugs acting on the autonomic nervous system, undetermined intent \| \| Y136 \| Poisoning by and exposure to other drugs acting on the autonomic nervous system, undetermined intent \| \| Y137 \| Poisoning by and exposure to other drugs acting on the autonomic nervous system, undetermined intent \| \| Y138 \| Poisoning by and exposure to other drugs acting on the autonomic nervous system, undetermined intent \| \| Y139 \| Poisoning by and exposure to other drugs acting on the autonomic nervous system, undetermined intent \| \| Y14 \| Poisoning by and exposure to other and unspecified drugs, medicaments and biological substances, undetermined intent \| \| Y140 \| Poisoning by and exposure to other and unspecified drugs, medicaments and biological substances, undetermined intent \| \| Y141 \| Poisoning by and exposure to other and unspecified drugs, medicaments and biological substances, undetermined intent \| \| Y142 \| Poisoning by and exposure to other and unspecified drugs, medicaments and biological substances, undetermined intent \| \| Y143 \| Poisoning by and exposure to other and unspecified drugs, medicaments and biological substances, undetermined intent \| \| Y144 \| Poisoning by and exposure to other and unspecified drugs, medicaments and biological substances, undetermined intent \| \| Y145 \| Poisoning by and exposure to other and unspecified drugs, medicaments and biological substances, undetermined intent \| \| Y146 \| Poisoning by and exposure to other and unspecified drugs, medicaments and biological substances, undetermined intent \| \| Y147 \| Poisoning by and exposure to other and unspecified drugs, medicaments and biological substances, undetermined intent \| \| Y148 \| Poisoning by and exposure to other and unspecified drugs, medicaments and biological substances, undetermined intent \| \| Y149 \| Poisoning by and exposure to other and unspecified drugs, medicaments and biological substances, undetermined intent \| \| Y15 \| Poisoning by and exposure to alcohol, undetermined intent \| \| Y150 \| Poisoning by and exposure to alcohol, undetermined intent \| \| Y151 \| Poisoning by and exposure to alcohol, undetermined intent \| \| Y152 \| Poisoning by and exposure to alcohol, undetermined intent \| \| Y153 \| Poisoning by and exposure to alcohol, undetermined intent \| \| Y154 \| Poisoning by and exposure to alcohol, undetermined intent \| \| Y155 \| Poisoning by and exposure to alcohol, undetermined intent \| \| Y156 \| Poisoning by and exposure to alcohol, undetermined intent \| \| Y157 \| Poisoning by and exposure to alcohol, undetermined intent \| \| Y158 \| Poisoning by and exposure to alcohol, undetermined intent \| \| Y159 \| Poisoning by and exposure to alcohol, undetermined intent \| \| Y16 \| Poisoning by and exposure to organic solvents and halogenated hydrocarbons and their vapours, undetermined intent \| \| Y160 \| Poisoning by and exposure to organic solvents and halogenated hydrocarbons and their vapours, undetermined intent \| \| Y161 \| Poisoning by and exposure to organic solvents and halogenated hydrocarbons and their vapours, undetermined intent \| \| Y162 \| Poisoning by and exposure to organic solvents and halogenated hydrocarbons and their vapours, undetermined intent \| \| Y163 \| Poisoning by and exposure to organic solvents and halogenated hydrocarbons and their vapours, undetermined intent \| \| Y164 \| Poisoning by and exposure to organic solvents and halogenated hydrocarbons and their vapours, undetermined intent \| \| Y165 \| Poisoning by and exposure to organic solvents and halogenated hydrocarbons and their vapours, undetermined intent \| \| Y166 \| Poisoning by and exposure to organic solvents and halogenated hydrocarbons and their vapours, undetermined intent \| \| Y167 \| Poisoning by and exposure to organic solvents and halogenated hydrocarbons and their vapours, undetermined intent \| \| Y168 \| Poisoning by and exposure to organic solvents and halogenated hydrocarbons and their vapours, undetermined intent \| \| Y169 \| Poisoning by and exposure to organic solvents and halogenated hydrocarbons and their vapours, undetermined intent \| \| Y17 \| Poisoning by and exposure to other gases and vapours, undetermined intent \| \| Y170 \| Poisoning by and exposure to other gases and vapours, undetermined intent \| \| Y171 \| Poisoning by and exposure to other gases and vapours, undetermined intent \| \| Y172 \| Poisoning by and exposure to other gases and vapours, undetermined intent \| \| Y173 \| Poisoning by and exposure to other gases and vapours, undetermined intent \| \| Y174 \| Poisoning by and exposure to other gases and vapours, undetermined intent \| \| Y175 \| Poisoning by and exposure to other gases and vapours, undetermined intent \| \| Y176 \| Poisoning by and exposure to other gases and vapours, undetermined intent \| \| Y177 \| Poisoning by and exposure to other gases and vapours, undetermined intent \| \| Y178 \| Poisoning by and exposure to other gases and vapours, undetermined intent \| \| Y179 \| Poisoning by and exposure to other gases and vapours, undetermined intent \| \| Y18 \| Poisoning by and exposure to pesticides, undetermined intent \| \| Y180 \| Poisoning by and exposure to pesticides, undetermined intent \| \| Y181 \| Poisoning by and exposure to pesticides, undetermined intent \| \| Y182 \| Poisoning by and exposure to pesticides, undetermined intent \| \| Y183 \| Poisoning by and exposure to pesticides, undetermined intent \| \| Y184 \| Poisoning by and exposure to pesticides, undetermined intent \| \| Y185 \| Poisoning by and exposure to pesticides, undetermined intent \| \| Y186 \| Poisoning by and exposure to pesticides, undetermined intent \| \| Y187 \| Poisoning by and exposure to pesticides, undetermined intent \| \| Y188 \| Poisoning by and exposure to pesticides, undetermined intent \| \| Y189 \| Poisoning by and exposure to pesticides, undetermined intent \| \| Y19 \| Poisoning by and exposure to other and unspecified chemicals and noxious substances, undetermined intent \| \| Y190 \| Poisoning by and exposure to other and unspecified chemicals and noxious substances, undetermined intent \| \| Y191 \| Poisoning by and exposure to other and unspecified chemicals and noxious substances, undetermined intent \| \| Y192 \| Poisoning by and exposure to other and unspecified chemicals and noxious substances, undetermined intent \| \| Y193 \| Poisoning by and exposure to other and unspecified chemicals and noxious substances, undetermined intent \| \| Y194 \| Poisoning by and exposure to other and unspecified chemicals and noxious substances, undetermined intent \| \| Y195 \| Poisoning by and exposure to other and unspecified chemicals and noxious substances, undetermined intent \| \| Y196 \| Poisoning by and exposure to other and unspecified chemicals and noxious substances, undetermined intent \| \| Y197 \| Poisoning by and exposure to other and unspecified chemicals and noxious substances, undetermined intent \| \| Y198 \| Poisoning by and exposure to other and unspecified chemicals and noxious substances, undetermined intent \| \| Y199 \| Poisoning by and exposure to other and unspecified chemicals and noxious substances, undetermined intent \| \| Y20 \| Hanging, strangulation and suffocation, undetermined intent \| \| Y200 \| Hanging, strangulation and suffocation, undetermined intent \| \| Y201 \| Hanging, strangulation and suffocation, undetermined intent \| \| Y202 \| Hanging, strangulation and suffocation, undetermined intent \| \| Y203 \| Hanging, strangulation and suffocation, undetermined intent \| \| Y204 \| Hanging, strangulation and suffocation, undetermined intent \| \| Y205 \| Hanging, strangulation and suffocation, undetermined intent \| \| Y206 \| Hanging, strangulation and suffocation, undetermined intent \| \| Y207 \| Hanging, strangulation and suffocation, undetermined intent \| \| Y208 \| Hanging, strangulation and suffocation, undetermined intent \| \| Y209 \| Hanging, strangulation and suffocation, undetermined intent \| \| Y21 \| Drowning and submersion, undetermined intent \| \| Y210 \| Drowning and submersion, undetermined intent \| \| Y211 \| Drowning and submersion, undetermined intent \| \| Y212 \| Drowning and submersion, undetermined intent \| \| Y213 \| Drowning and submersion, undetermined intent \| \| Y214 \| Drowning and submersion, undetermined intent \| \| Y215 \| Drowning and submersion, undetermined intent \| \| Y216 \| Drowning and submersion, undetermined intent \| \| Y217 \| Drowning and submersion, undetermined intent \| \| Y218 \| Drowning and submersion, undetermined intent \| \| Y219 \| Drowning and submersion, undetermined intent \| \| Y22 \| Handgun discharge, undetermined intent \| \| Y220 \| Handgun discharge, undetermined intent \| \| Y221 \| Handgun discharge, undetermined intent \| \| Y222 \| Handgun discharge, undetermined intent \| \| Y223 \| Handgun discharge, undetermined intent \| \| Y224 \| Handgun discharge, undetermined intent \| \| Y225 \| Handgun discharge, undetermined intent \| \| Y226 \| Handgun discharge, undetermined intent \| \| Y227 \| Handgun discharge, undetermined intent \| \| Y228 \| Handgun discharge, undetermined intent \| \| Y229 \| Handgun discharge, undetermined intent \| \| Y23 \| Rifle, shotgun and larger firearm discharge, undetermined intent \| \| Y230 \| Rifle, shotgun and larger firearm discharge, undetermined intent \| \| Y231 \| Rifle, shotgun and larger firearm discharge, undetermined intent \| \| Y232 \| Rifle, shotgun and larger firearm discharge, undetermined intent \| \| Y233 \| Rifle, shotgun and larger firearm discharge, undetermined intent \| \| Y234 \| Rifle, shotgun and larger firearm discharge, undetermined intent \| \| Y235 \| Rifle, shotgun and larger firearm discharge, undetermined intent \| \| Y236 \| Rifle, shotgun and larger firearm discharge, undetermined intent \| \| Y237 \| Rifle, shotgun and larger firearm discharge, undetermined intent \| \| Y238 \| Rifle, shotgun and larger firearm discharge, undetermined intent \| \| Y239 \| Rifle, shotgun and larger firearm discharge, undetermined intent \| \| Y24 \| Other and unspecified firearm discharge, undetermined intent \| \| Y240 \| Other and unspecified firearm discharge, undetermined intent \| \| Y241 \| Other and unspecified firearm discharge, undetermined intent \| \| Y242 \| Other and unspecified firearm discharge, undetermined intent \| \| Y243 \| Other and unspecified firearm discharge, undetermined intent \| \| Y244 \| Other and unspecified firearm discharge, undetermined intent \| \| Y245 \| Other and unspecified firearm discharge, undetermined intent \| \| Y246 \| Other and unspecified firearm discharge, undetermined intent \| \| Y247 \| Other and unspecified firearm discharge, undetermined intent \| \| Y248 \| Other and unspecified firearm discharge, undetermined intent \| \| Y249 \| Other and unspecified firearm discharge, undetermined intent \| \| Y25 \| Contact with explosive material, undetermined intent \| \| Y250 \| Contact with explosive material, undetermined intent \| \| Y251 \| Contact with explosive material, undetermined intent \| \| Y252 \| Contact with explosive material, undetermined intent \| \| Y253 \| Contact with explosive material, undetermined intent \| \| Y254 \| Contact with explosive material, undetermined intent \| \| Y255 \| Contact with explosive material, undetermined intent \| \| Y256 \| Contact with explosive material, undetermined intent \| \| Y257 \| Contact with explosive material, undetermined intent \| \| Y258 \| Contact with explosive material, undetermined intent \| \| Y259 \| Contact with explosive material, undetermined intent \| \| Y26 \| Exposure to smoke, fire and flames, undetermined intent \| \| Y260 \| Exposure to smoke, fire and flames, undetermined intent \| \| Y261 \| Exposure to smoke, fire and flames, undetermined intent \| \| Y262 \| Exposure to smoke, fire and flames, undetermined intent \| \| Y263 \| Exposure to smoke, fire and flames, undetermined intent \| \| Y264 \| Exposure to smoke, fire and flames, undetermined intent \| \| Y265 \| Exposure to smoke, fire and flames, undetermined intent \| \| Y266 \| Exposure to smoke, fire and flames, undetermined intent \| \| Y267 \| Exposure to smoke, fire and flames, undetermined intent \| \| Y268 \| Exposure to smoke, fire and flames, undetermined intent \| \| Y269 \| Exposure to smoke, fire and flames, undetermined intent \| \| Y27 \| Contact with steam, hot vapours and hot objects, undetermined intent \| \| Y270 \| Contact with steam, hot vapours and hot objects, undetermined intent \| \| Y271 \| Contact with steam, hot vapours and hot objects, undetermined intent \| \| Y272 \| Contact with steam, hot vapours and hot objects, undetermined intent \| \| Y273 \| Contact with steam, hot vapours and hot objects, undetermined intent \| \| Y274 \| Contact with steam, hot vapours and hot objects, undetermined intent \| \| Y275 \| Contact with steam, hot vapours and hot objects, undetermined intent \| \| Y276 \| Contact with steam, hot vapours and hot objects, undetermined intent \| \| Y277 \| Contact with steam, hot vapours and hot objects, undetermined intent \| \| Y278 \| Contact with steam, hot vapours and hot objects, undetermined intent \| \| Y279 \| Contact with steam, hot vapours and hot objects, undetermined intent \| \| Y28 \| Contact with sharp object, undetermined intent \| \| Y280 \| Contact with sharp object, undetermined intent \| \| Y281 \| Contact with sharp object, undetermined intent \| \| Y282 \| Contact with sharp object, undetermined intent \| \| Y283 \| Contact with sharp object, undetermined intent \| \| Y284 \| Contact with sharp object, undetermined intent \| \| Y285 \| Contact with sharp object, undetermined intent \| \| Y286 \| Contact with sharp object, undetermined intent \| \| Y287 \| Contact with sharp object, undetermined intent \| \| Y288 \| Contact with sharp object, undetermined intent \| \| Y289 \| Contact with sharp object, undetermined intent \| \| Y29 \| Contact with blunt object, undetermined intent \| \| Y290 \| Contact with blunt object, undetermined intent \| \| Y291 \| Contact with blunt object, undetermined intent \| \| Y292 \| Contact with blunt object, undetermined intent \| \| Y293 \| Contact with blunt object, undetermined intent \| \| Y294 \| Contact with blunt object, undetermined intent \| \| Y295 \| Contact with blunt object, undetermined intent \| \| Y296 \| Contact with blunt object, undetermined intent \| \| Y297 \| Contact with blunt object, undetermined intent \| \| Y298 \| Contact with blunt object, undetermined intent \| \| Y299 \| Contact with blunt object, undetermined intent \| \| Y30 \| Falling, jumping or pushed from a high place, undetermined intent \| \| Y300 \| Falling, jumping or pushed from a high place, undetermined intent \| \| Y301 \| Falling, jumping or pushed from a high place, undetermined intent \| \| Y302 \| Falling, jumping or pushed from a high place, undetermined intent \| \| Y303 \| Falling, jumping or pushed from a high place, undetermined intent \| \| Y304 \| Falling, jumping or pushed from a high place, undetermined intent \| \| Y305 \| Falling, jumping or pushed from a high place, undetermined intent \| \| Y306 \| Falling, jumping or pushed from a high place, undetermined intent \| \| Y307 \| Falling, jumping or pushed from a high place, undetermined intent \| \| Y308 \| Falling, jumping or pushed from a high place, undetermined intent \| \| Y309 \| Falling, jumping or pushed from a high place, undetermined intent \| \| Y31 \| Falling, lying or running before or into moving object, undetermined intent \| \| Y310 \| Falling, lying or running before or into moving object, undetermined intent \| \| Y311 \| Falling, lying or running before or into moving object, undetermined intent \| \| Y312 \| Falling, lying or running before or into moving object, undetermined intent \| \| Y313 \| Falling, lying or running before or into moving object, undetermined intent \| \| Y314 \| Falling, lying or running before or into moving object, undetermined intent \| \| Y315 \| Falling, lying or running before or into moving object, undetermined intent \| \| Y316 \| Falling, lying or running before or into moving object, undetermined intent \| \| Y317 \| Falling, lying or running before or into moving object, undetermined intent \| \| Y318 \| Falling, lying or running before or into moving object, undetermined intent \| \| Y319 \| Falling, lying or running before or into moving object, undetermined intent \| \| Y32 \| Crashing of motor vehicle, undetermined intent \| \| Y320 \| Crashing of motor vehicle, undetermined intent \| \| Y321 \| Crashing of motor vehicle, undetermined intent \| \| Y322 \| Crashing of motor vehicle, undetermined intent \| \| Y323 \| Crashing of motor vehicle, undetermined intent \| \| Y324 \| Crashing of motor vehicle, undetermined intent \| \| Y325 \| Crashing of motor vehicle, undetermined intent \| \| Y326 \| Crashing of motor vehicle, undetermined intent \| \| Y327 \| Crashing of motor vehicle, undetermined intent \| \| Y328 \| Crashing of motor vehicle, undetermined intent \| \| Y329 \| Crashing of motor vehicle, undetermined intent \| \| Y33 \| Other specified events, undetermined intent \| \| Y330 \| Other specified events, undetermined intent \| \| Y331 \| Other specified events, undetermined intent \| \| Y332 \| Other specified events, undetermined intent \| \| Y333 \| Other specified events, undetermined intent \| \| Y334 \| Other specified events, undetermined intent \| \| Y335 \| Other specified events, undetermined intent \| \| Y336 \| Other specified events, undetermined intent \| \| Y337 \| Other specified events, undetermined intent \| \| Y338 \| Other specified events, undetermined intent \| \| Y339 \| Other specified events, undetermined intent \| \| Y34 \| Unspecified event, undetermined intent \| \| Y340 \| Unspecified event, undetermined intent \| \| Y341 \| Unspecified event, undetermined intent \| \| Y342 \| Unspecified event, undetermined intent \| \| Y343 \| Unspecified event, undetermined intent \| \| Y344 \| Unspecified event, undetermined intent \| \| Y345 \| Unspecified event, undetermined intent \| \| Y346 \| Unspecified event, undetermined intent \| \| Y347 \| Unspecified event, undetermined intent \| \| Y348 \| Unspecified event, undetermined intent \| \| Y349 \| Unspecified event, undetermined intent \| \| Y870 \| Sequelae of intentional self-harm \| \| Y872 \| Sequelae of events of undetermined intent \| |
| **GP: ANTIDEPRESSANT** |
| \| 66580 \| Antidepressant drug treatment changed \| \| --- \| --- \| \| 66590 \| Antidepressant drug treatment started \| \| 665A0 \| Antidepressant drug treatment stopped \| \| d7... \| TRICYCLIC ANTIDEPRESSANTS \| \| d71.. \| AMITRIPTYLINE HYDROCHLORIDE [ANTIDEPRESSANT] \| \| d711. \| AMITRIPTYLINE 10mg tablets \| \| d712. \| AMITRIPTYLINE 25mg tablets \| \| d713. \| AMITRIPTYLINE 50mg tablets \| \| d714. \| *DOMICAL 10mg tablets \| \| d715. \| *DOMICAL 25mg tablets \| \| d716. \| *DOMICAL 50mg tablets \| \| d717. \| ELAVIL 10mg tablets \| \| d718. \| ELAVIL 25mg tablets \| \| d719. \| *LENTIZOL 25mg m/r capsules \| \| d71a. \| *LENTIZOL 50mg m/r capsules \| \| d71b. \| *TRYPTIZOL 75mg m/r capsules \| \| d71c. \| *TRYPTIZOL 10mg tablets \| \| d71d. \| *TRYPTIZOL 25mg tablets \| \| d71e. \| *TRYPTIZOL 50mg tablets \| \| d71f. \| *TRYPTIZOL 10mg/5mL syrup \| \| d71g. \| TRYPTIZOL 100mg/10mL injection \| \| d71h. \| AMITRIPTYLINE 25mg/5mL sugar free solution \| \| d71i. \| AMITRIPTYLINE 50mg/5mL sugar free solution \| \| d71u. \| AMITRIPTYLINE 25mg m/r capsules \| \| d71v. \| AMITRIPTYLINE 50mg m/r capsules \| \| d71w. \| AMITRIPTYLINE 75mg m/r capsules \| \| d71y. \| *AMITRIPTYLINE 10mg/5mL syrup \| \| d71z. \| AMITRIPTYLINE HYDROCHLORIDE 100mg/10mL injection \| \| d72.. \| *BUTRIPTYLINE \| \| d721. \| *EVADYNE 25mg tablets \| \| d722. \| *EVADYNE 50mg tablets \| \| d72y. \| *BUTRIPTYLINE 25mg tablets \| \| d72z. \| *BUTRIPTYLINE 50mg tablets \| \| d73.. \| CLOMIPRAMINE HYDROCHLORIDE \| \| d731. \| ANAFRANIL 10mg capsules \| \| d732. \| ANAFRANIL 25mg capsules \| \| d733. \| ANAFRANIL 50mg capsules \| \| d734. \| *ANAFRANIL 25mg/5mL syrup \| \| d735. \| *ANAFRANIL 25mg/2mL injection \| \| d736. \| ANAFRANIL SR 75mg m/r tablets \| \| d737. \| *TRANQUAX 10mg capsules \| \| d738. \| *TRANQUAX 25mg capsules \| \| d739. \| TRANQUAX 50mg capsules \| \| d73r. \| *CLOMIPRAMINE HCL 50mg tablets \| \| d73s. \| *CLOMIPRAMINE HCL 10mg tablets \| \| d73t. \| *CLOMIPRAMINE HCL 25mg tablets \| \| d73u. \| CLOMIPRAMINE HCL 10mg capsules \| \| d73v. \| CLOMIPRAMINE HCL 25mg capsules \| \| d73w. \| CLOMIPRAMINE HCL 50mg capsules \| \| d73x. \| CLOMIPRAMINE HYDROCHLORIDE 25mg/5mL syrup \| \| d73y. \| CLOMIPRAMINE HCL 25mg/2mL injection \| \| d73z. \| CLOMIPRAMINE HCL 75mg m/r tabs \| \| d74.. \| DESIPRAMINE HYDROCHLORIDE \| \| d741. \| *PERTOFRAN 25mg tablets \| \| d74z. \| DESIPRAMINE HYDROCHLORIDE 25mg tablets \| \| d75.. \| DOSULEPIN HYDROCHLORIDE \| \| d751. \| PROTHIADEN 25mg capsules \| \| d752. \| PROTHIADEN 75mg tablets \| \| d753. \| *PREPADINE 25mg capsules \| \| d754. \| *PREPADINE 75mg tablets \| \| d755. \| *DOTHAPAX 25mg capsules \| \| d756. \| *DOTHAPAX 75mg tablets \| \| d759. \| *THADEN 25mg capsules \| \| d75A. \| *THADEN 75mg tablets \| \| d75y. \| DOSULEPIN HYDROCHLORIDE 25mg capsules \| \| d75z. \| DOSULEPIN HYDROCHLORIDE 75mg tablets \| \| d76.. \| DOXEPIN \| \| d761. \| *SINEQUAN 10mg capsules x56CP \| \| d762. \| *SINEQUAN 25mg capsules x28CP \| \| d763. \| *SINEQUAN 50mg capsules x28CP \| \| d764. \| *SINEQUAN 75mg capsules x28CP \| \| d765. \| SINEPIN 25mg capsules \| \| d766. \| SINEPIN 50mg capsules \| \| d76w. \| *DOXEPIN 10mg capsules \| \| d76x. \| DOXEPIN 25mg capsules \| \| d76y. \| DOXEPIN 50mg capsules \| \| d76z. \| *DOXEPIN 75mg capsules \| \| d77.. \| IMIPRAMINE HYDROCHLORIDE [ANTIDEPRESSANT] \| \| d771. \| IMIPRAMINE 10mg tabs \| \| d772. \| IMIPRAMINE 25mg tablets \| \| d773. \| *PRAMINIL 10mg tablets \| \| d774. \| *PRAMINIL 25mg tablets \| \| d775. \| *TOFRANIL 10mg tablets \| \| d776. \| *TOFRANIL 25mg tablets \| \| d777. \| *TOFRANIL 25mg/5mL syrup \| \| d77w. \| IMIPRAMINE HYDROCHLORIDE 25mg/5mL oral solution \| \| d77x. \| IMIPRAMINE HYDROCHLORIDE 10mg tablets \| \| d77y. \| IMIPRAMINE HYDROCHLORIDE 25mg tablets \| \| d77z. \| IMIPRAMINE HYDROCHLORIDE 25mg/5mL syrup \| \| d78.. \| IPRINDOLE \| \| d781. \| *PRONDOL 15mg tablets \| \| d782. \| *PRONDOL 30mg tablets \| \| d78y. \| *IPRINDOLE 15mg tablets \| \| d78z. \| *IPRINDOLE 30mg tablets \| \| d79.. \| LOFEPRAMINE \| \| d791. \| *GAMANIL 70mg tablets x56CP \| \| d792. \| LOMONT 70mg/5mL sugar free suspension \| \| d793. \| *FEPRAPAX 70mg tablets \| \| d794. \| *GAMANIL 70mg tablets \| \| d79y. \| LOFEPRAMINE 70mg/5mL sugar free suspension \| \| d79z. \| LOFEPRAMINE 70mg tablets \| \| d7a.. \| MAPROTILINE HYDROCHLORIDE \| \| d7a1. \| *LUDIOMIL 10mg tablets \| \| d7a2. \| *LUDIOMIL 25mg tablets \| \| d7a3. \| *LUDIOMIL 50mg tablets \| \| d7a4. \| *LUDIOMIL 75mg tablets x28CP \| \| d7aw. \| MAPROTILINE HYDROCHLORIDE 10mg tablets \| \| d7ax. \| MAPROTILINE HYDROCHLORIDE 25mg tablets \| \| d7ay. \| MAPROTILINE HYDROCHLORIDE 50mg tablets \| \| d7az. \| MAPROTILINE HYDROCHLORIDE 75mg tablets \| \| d7b.. \| MIANSERIN HYDROCHLORIDE \| \| d7b1. \| MIANSERIN 10mg tablets \| \| d7b2. \| MIANSERIN 20mg tablets \| \| d7b3. \| MIANSERIN 30mg tablets \| \| d7b4. \| *BOLVIDON 10mg tablets \| \| d7b5. \| *BOLVIDON 20mg tablets \| \| d7b6. \| *BOLVIDON 30mg tablets \| \| d7b7. \| *NORVAL 10mg tablets \| \| d7b8. \| *NORVAL 20mg tablets \| \| d7b9. \| *NORVAL 30mg tablets \| \| d7c.. \| NORTRIPTYLINE \| \| d7c1. \| *ALLEGRON 10mg tablets \| \| d7c2. \| *ALLEGRON 25mg tablets \| \| d7c3. \| *AVENTYL 10mg capsules \| \| d7c4. \| *AVENTYL 25mg capsules \| \| d7c5. \| *AVENTYL 10mg/5mL liquid \| \| d7c6. \| NORTRIPTYLINE 10mg tablets \| \| d7c7. \| *NORTRIPTYLINE 10mg/5mL liquid \| \| d7c8. \| NORTRIPTYLINE 25mg tablets \| \| d7c9. \| *NORTRIPTYLINE 10mg capsules \| \| d7cy. \| *NORTRIPTYLINE 25mg capsules \| \| d7d.. \| PROTRIPTYLINE HYDROCHLORIDE \| \| d7d1. \| *CONCORDIN 5mg tablets \| \| d7d2. \| *CONCORDIN 10mg tablets \| \| d7d3. \| PROTRIPTYLINE HYDROCHLORIDE 5mg tablets \| \| d7d4. \| PROTRIPTYLINE HYDROCHLORIDE 10mg tablets \| \| d7e.. \| TRAZODONE HYDROCHLORIDE \| \| d7e1. \| MOLIPAXIN 50mg capsules x84CP \| \| d7e2. \| MOLIPAXIN 100mg capsules x56CP \| \| d7e3. \| *MOLIPAXIN 50mg/5mL liquid \| \| d7e4. \| MOLIPAXIN 150mg tablets x28CP \| \| d7e5. \| TRAZODONE HYDROCHLORIDE 150mg tablets \| \| d7e6. \| MOLIPAXIN CR 150mg m/r tablets x28 \| \| d7e7. \| TRAZODONE HYDROCHLORIDE 150mg m/r tablets \| \| d7ew. \| TRAZODONE HYDROCHLORIDE 100mg capsules \| \| d7ex. \| TRAZODONE HYDROCHLORIDE 50mg capsules \| \| d7ez. \| TRAZODONE HYDROCHLORIDE 50mg/5mL liquid \| \| d7f.. \| TRIMIPRAMINE \| \| d7f1. \| SURMONTIL 50mg capsules x28CP \| \| d7f2. \| SURMONTIL 10mg tablets \| \| d7f3. \| SURMONTIL 25mg tablets \| \| d7fx. \| TRIMIPRAMINE 50mg capsules \| \| d7fy. \| TRIMIPRAMINE 10mg tablets \| \| d7fz. \| TRIMIPRAMINE 25mg tablets \| \| d7g.. \| VILOXAZINE HYDROCHLORIDE \| \| d7g1. \| *VIVALAN 50mg tablets \| \| d7gz. \| *VILOXAZINE 50mg tablets \| \| d7h.. \| AMOXAPINE \| \| d7h1. \| *AMOXAPINE 25mg tablets \| \| d7h2. \| *AMOXAPINE 50mg tablets \| \| d7h3. \| *AMOXAPINE 100mg tablets \| \| d7h4. \| *AMOXAPINE 150mg tablets \| \| d7h5. \| *ASENDIS 25mg tablets \| \| d7h6. \| *ASENDIS 50mg tablets \| \| d7h7. \| *ASENDIS 100mg tablets \| \| d7h8. \| *ASENDIS 150mg tablets \| \| d9... \| COMPOUND ANTIDEPRESSANT DRUGS \| \| d91.. \| COMPOUND ANTIDEPRESSANTS A-Z \| \| d911. \| *LIMBITROL 5 capsules \| \| d912. \| *LIMBITROL 10 capsules \| \| d913. \| *MOTIPRESS tablets x28CP \| \| d914. \| *MOTIVAL tablets \| \| d915. \| *PARSTELIN tablets \| \| d916. \| TRIPTAFEN tablets \| \| d917. \| *TRIPTAFEN-M tablets \| \| da... \| OTHER ANTIDEPRESSANT DRUGS \| \| da1.. \| FLUPENTIXOL [ANTIDEPRESSANT] \| \| da11. \| FLUANXOL 500micrograms tablets \| \| da12. \| FLUANXOL 1mg tablets \| \| da1y. \| FLUPENTIXOL 500micrograms tablets \| \| da1z. \| FLUPENTIXOL 1mg tablets \| \| da2.. \| TRYPTOPHAN \| \| da21. \| OPTIMAX 500mg tablets \| \| da22. \| *OPTIMAX 1g/6g powder \| \| da23. \| *OPTIMAX WV 500mg tablets \| \| da24. \| *PACITRON 500mg tablets \| \| da2y. \| TRYPTOPHAN 500mg tablets \| \| da2z. \| *TRYPTOPHAN 1g/6g powder \| \| da3.. \| FLUVOXAMINE MALEATE \| \| da31. \| FAVERIN 50mg tablets \| \| da32. \| FLUVOXAMINE MALEATE 50mg tablets \| \| da33. \| FAVERIN 100mg tablets \| \| da34. \| FLUVOXAMINE MALEATE 100mg tablets \| \| da4.. \| FLUOXETINE HYDROCHLORIDE \| \| da41. \| FLUOXETINE 20mg capsules \| \| da42. \| *PROZAC 20mg capsules x30 \| \| da43. \| FLUOXETINE 20mg/5mL oral liquid \| \| da44. \| PROZAC 20mg/5mL oral liquid \| \| da45. \| PROZAC 20mg capsules \| \| da46. \| FLUOXETINE 60mg capsules \| \| da47. \| *PROZAC 60mg capsules \| \| da48. \| *FELICIUM 20mg capsules \| \| da49. \| OXACTIN 20mg capsules \| \| da4A. \| RANFLUTIN 20mg capsules \| \| da4B. \| PROZIT 20mg/5mL oral solution \| \| da4C. \| PROZEP 20mg/5mL oral solution \| \| da5.. \| SERTRALINE HYDROCHLORIDE \| \| da51. \| SERTRALINE 50mg tablets \| \| da52. \| SERTRALINE 100mg tablets \| \| da53. \| LUSTRAL 50mg tablets \| \| da54. \| LUSTRAL 100mg tablets \| \| da6.. \| PAROXETINE HYDROCHLORIDE \| \| da61. \| PAROXETINE 20mg tablets \| \| da62. \| SEROXAT 20mg tablets x30 \| \| da63. \| PAROXETINE 30mg tablets \| \| da64. \| SEROXAT 30mg tablets x30 \| \| da65. \| PAROXETINE 10mg/5mL sugar free liquid \| \| da66. \| SEROXAT 10mg/5mL sugar free liquid \| \| da67. \| PAROXETINE 10mg tablets \| \| da68. \| SEROXAT 10mg tablets \| \| da7.. \| VENLAFAXINE \| \| da71. \| VENLAFAXINE 37.5mg tablets \| \| da72. \| VENLAFAXINE 75mg tablets \| \| da73. \| *EFEXOR 37.5mg tablets \| \| da74. \| *EFEXOR 75mg tablets \| \| da75. \| *VENLAFAXINE 50mg tablets \| \| da76. \| *EFEXOR 50mg tablets \| \| da77. \| VENLAFAXINE 75mg m/r capsules \| \| da78. \| EFEXOR XL 75mg m/r capsules \| \| da79. \| VENLAFAXINE 150mg m/r capsules \| \| da7a. \| VENAXX XL 150mg m/r capsules \| \| da7A. \| EFEXOR XL 150mg m/r capsules \| \| da7b. \| VAXALIN XL 75mg m/r capsules \| \| da7B. \| RODOMEL XL 75mg m/r capsules \| \| da7c. \| VAXALIN XL 150mg m/r capsules \| \| da7C. \| RODOMEL XL 150mg m/r capsules \| \| da7d. \| ALVENTA XL 75mg m/r capsules \| \| da7D. \| WINFEX XL 75mg m/r capsules \| \| da7e. \| ALVENTA XL 150mg m/r capsules \| \| da7E. \| WINFEX XL 150mg m/r capsules \| \| da7f. \| RANFAXINE XL 150mg m/r capsules \| \| da7F. \| TRIXAT XL 75mg m/r capsules \| \| da7g. \| RANFAXINE XL 75mg m/r capsules \| \| da7G. \| TRIXAT XL 150mg m/r capsules \| \| da7h. \| BONILUX XL 75mg m/r capsules \| \| da7H. \| VIEPAX XL 75mg m/r tablets \| \| da7i. \| BONILUX XL 150mg m/r capsules \| \| da7I. \| VENLAFAXINE 75mg m/r tablets \| \| da7j. \| TONPULAR XL 75mg m/r capsules \| \| da7J. \| VIEPAX XL 150mg m/r tablets \| \| da7k. \| TONPULAR XL 150mg m/r capsules \| \| da7K. \| VENLAFAXINE 150mg m/r tablets \| \| da7l. \| FORAVEN XL 75mg m/r capsules \| \| da7L. \| *TARDCAPS XL 75mg m/r capsules \| \| da7m. \| FORAVEN XL 150mg m/r capsules \| \| da7M. \| *TARDCAPS XL 150mg m/r capsule \| \| da7n. \| DEPEFEX XL 75mg m/r capsules \| \| da7N. \| VIEPAX 37.5mg tablets \| \| da7o. \| DEPEFEX XL 150mg m/r capsules \| \| da7O. \| VIEPAX 75mg tablets \| \| da7p. \| VENLALIC XL 37.5mg m/r tablets \| \| da7P. \| VENSIR XL 75mg m/r capsules \| \| da7q. \| VENLAFAXINE 37.5mg m/r tablets \| \| da7Q. \| VENSIR XL 150mg m/r capsules \| \| da7R. \| TIFAXIN XL 75mg m/r capsules \| \| da7S. \| TIFAXIN XL 150mg m/r capsules \| \| da7T. \| VEXARIN XL 75mg m/r capsules \| \| da7U. \| VEXARIN XL 150mg m/r capsules \| \| da7V. \| VENLALIC XL 75mg m/r tablets \| \| da7W. \| VENLALIC XL 150mg m/r tablets \| \| da7X. \| VENLALIC XL 225mg m/r tablets \| \| da7Y. \| VENLAFAXINE 225mg m/r tablets \| \| da7Z. \| VENAXX XL 75mg m/r capsules \| \| da8.. \| NEFAZODONE \| \| da81. \| NEFAZODONE HYDROCHLORIDE 100mg tablets \| \| da82. \| NEFAZODONE HYDROCHLORIDE 200mg tablets \| \| da83. \| *DUTONIN 100mg tablets \| \| da84. \| *DUTONIN 200mg tablets \| \| da85. \| NEFAZODONE HCL 50mg+100mg+200mg initiation tablets pack \| \| da86. \| DUTONIN 50mg+100mg+200mg treatment initiation tablets pack \| \| da9.. \| CITALOPRAM \| \| da91. \| CITALOPRAM 20mg tablets \| \| da92. \| CIPRAMIL 20mg tablets \| \| da93. \| CITALOPRAM 10mg tablets \| \| da94. \| CIPRAMIL 10mg tablets \| \| da95. \| CITALOPRAM 40mg tablets \| \| da96. \| CIPRAMIL 40mg tablets \| \| da97. \| CIPRAMIL 40mg/mL oral drops 15mL \| \| da98. \| *PAXORAN 10mg tablets \| \| da99. \| *PAXORAN 20mg tablets \| \| da9A. \| *PAXORAN 40mg tablets \| \| da9z. \| CITALOPRAM 40mg/mL oral drops \| \| daA.. \| REBOXETINE \| \| daA1. \| REBOXETINE 4mg tablets \| \| daA2. \| EDRONAX 4mg tablets \| \| daB.. \| MIRTAZAPINE \| \| daB1. \| MIRTAZAPINE 30mg tablets \| \| daB2. \| *ZISPIN 30mg tablets \| \| daB3. \| MIRTAZAPINE 30mg oro-dispersible tablets \| \| daB4. \| ZISPIN SOLTAB 30mg oro-dispersible tablets \| \| daB5. \| MIRTAZAPINE 15mg oro-dispersible tablets \| \| daB6. \| ZISPIN SOLTAB 15mg oro-dispersible tablets \| \| daB7. \| MIRTAZAPINE 45mg oro-dispersible tablets \| \| daB8. \| ZISPIN SOLTAB 45mg oro-dispersible tablets \| \| daBy. \| MIRTAZAPINE 45mg tablets \| \| daBz. \| MIRTAZAPINE 15mg tablets \| \| daC.. \| ESCITALOPRAM \| \| daC1. \| ESCITALOPRAM 10mg tablets \| \| daC2. \| CIPRALEX 10mg tablets \| \| daC3. \| ESCITALOPRAM 20mg tablets \| \| daC4. \| CIPRALEX 20mg tablets \| \| daC5. \| ESCITALOPRAM 5mg tablets \| \| daC6. \| CIPRALEX 5mg tablets \| \| daC7. \| ESCITALOPRAM 10mg/mL oral drops \| \| daC8. \| *CIPRALEX 10mg/mL oral drops \| \| daC9. \| CIPRALEX 20mg/mL oral drops \| \| daCA. \| ESCITALOPRAM 20mg/mL oral drops \| \| daD.. \| AGOMELATINE \| \| daD1. \| VALDOXAN 25mg tablets \| \| daD2. \| AGOMELATINE 25mg tablets \| \| E258. \| Nondependent antidepressant type drug abuse \| \| E2580 \| Nondependent antidepressant type drug abuse, unspecified \| \| E2581 \| Nondependent antidepressant type drug abuse, continuous \| \| E2582 \| Nondependent antidepressant type drug abuse, episodic \| \| E2583 \| Nondependent antidepressant type drug abuse in remission \| \| E258z \| Nondependent antidepressant type drug abuse NOS \| \| SL90. \| Antidepressant poisoning \| \| SL90z \| Anti-depressant poisoning NOS \| \| SyuFK \| [X]Poisoning by other and unspecified antidepressants \| \| T840. \| Accidental poisoning by antidepressants \| \| T840z \| Accidental poisoning by antidepressants NOS \| \| TJ90. \| Adverse reaction to antidepressants \| \| TJ90z \| Adverse reaction to antidepressants NOS \| \| U6090 \| [X]Tricyc/tetracyc antidepres caus advers eff therapeut use \| \| U6091 \| [X]Monoamine oxid inhib antidep caus advers eff therap use \| \| U6092 \| [X]Other unspec antidepres caus advers effect in therap use \| |
| **GP: ANTIHYPNOTICS** |
| \| d1... \| HYPNOTICS \| \| --- \| --- \| \| d11.. \| CHLORAL HYDRATE \| \| d111. \| *CHLORAL 500mg/5mL mixture \| \| d112. \| CHLORAL PAEDIATRIC 200mg/5mL elixir \| \| d113. \| *NOCTEC 500mg capsules \| \| d114. \| CHLORAL HYDRATE 500mg capsules \| \| d115. \| WELLDORM 143mg/5mL elixir \| \| d116. \| WELLDORM tablets \| \| d117. \| CHLORAL HYDRATE 414mg tablets \| \| d118. \| CHLORAL HYDRATE 143mg/5mL elixir \| \| d119. \| CHLORAL HYDRATE 500mg/5mL syrup \| \| d11A. \| SOMNWELL 707mg tablets \| \| d12.. \| CLOMETHIAZOLE EDISYLATE [HYPNOTIC] \| \| d121. \| *HEMINEVRIN 192mg capsules \| \| d122. \| *HEMINEVRIN 250mg/5mL syrup \| \| d123. \| *HEMINEVRIN 8mg/mL infusion \| \| d12v. \| CLOMETHIAZOLE 192mg capsules \| \| d12w. \| *CLOMETHIAZOLE 250mg/5mL syrup \| \| d12z. \| CHLORMETHIAZOLE EDISYLATE 8mg/mL infusion \| \| d13.. \| *DICHLORALPHENAZONE \| \| d131. \| *WELLDORM 650mg tablets \| \| d132. \| *WELLDORM 225mg/5mL elixir \| \| d13y. \| DICHLORALPHENAZONE 650mg tablets \| \| d13z. \| DICHLORALPHENAZONE 225mg/5mL elixir \| \| d14.. \| *FLUNITRAZEPAM \| \| d141. \| *ROHYPNOL 1mg tablets \| \| d14z. \| *FLUNITRAZEPAM 1mg tablets \| \| d15.. \| FLURAZEPAM \| \| d151. \| DALMANE 15mg capsules \| \| d152. \| DALMANE 30mg capsules \| \| d153. \| *PAXANE 15mg capsules \| \| d154. \| *PAXANE 30mg capsules \| \| d15y. \| FLURAZEPAM 15mg capsules \| \| d15z. \| FLURAZEPAM 30mg capsules \| \| d16.. \| LOPRAZOLAM \| \| d161. \| LOPRAZOLAM 1mg tablets \| \| d162. \| *DORMONOCT 1mg tablets \| \| d17.. \| LORMETAZEPAM \| \| d171. \| LORMETAZEPAM 500micrograms tablets \| \| d172. \| LORMETAZEPAM 1mg tablets \| \| d173. \| NOCTAMID 500micrograms tablets \| \| d174. \| *NOCTAMID 1mg tablets \| \| d18.. \| NITRAZEPAM \| \| d181. \| *NITRAZEPAM 5mg capsules \| \| d182. \| NITRAZEPAM 5mg tablets \| \| d183. \| *NITRAZEPAM 10mg tablets \| \| d184. \| NITRAZEPAM 2.5mg/5mL mixture \| \| d185. \| *MOGADON 5mg capsules \| \| d186. \| MOGADON 5mg tablets \| \| d187. \| *NITRADOS 5mg tablets \| \| d188. \| *NOCTESED 5mg tablets \| \| d189. \| REMNOS 5mg tablets \| \| d18a. \| *REMNOS 10mg tablets \| \| d18b. \| *SOMNITE 5mg tablets \| \| d18c. \| SOMNITE 2.5mg/5mL mixture \| \| d18d. \| *SUREM 5mg capsules \| \| d18e. \| *UNISOMNIA 5mg tablets \| \| d18f. \| NITRAZEPAM 5mg/5mL suspension \| \| d19.. \| PROMETHAZINE HCL [HYPNOTIC] see section c8i.. \| \| d1a.. \| TEMAZEPAM [HYPNOTIC] \| \| d1a1. \| *TEMAZEPAM 10mg capsules \| \| d1a2. \| *TEMAZEPAM 15mg capsules \| \| d1a3. \| *TEMAZEPAM 20mg capsules \| \| d1a4. \| *TEMAZEPAM 30mg capsules \| \| d1a5. \| *TEMAZEPAM 10mg/5mL elixir \| \| d1a6. \| *NORMISON 10mg capsules \| \| d1a7. \| *NORMISON 20mg capsules \| \| d1a8. \| *TEMAZEPAM PLANPAK capsules \| \| d1a9. \| TEMAZEPAM 10mg tablets \| \| d1aa. \| TEMAZEPAM 20mg tablets \| \| d1ab. \| TEMAZEPAM GELTHIX 10mg capsules \| \| d1ac. \| TEMAZEPAM GELTHIX 20mg capsules \| \| d1ad. \| TEMAZEPAM GELTHIX 15mg capsules \| \| d1ae. \| TEMAZEPAM GELTHIX 30mg capsules \| \| d1af. \| TEMAZEPAM GEL FILLED 10mg capsules \| \| d1ag. \| TEMAZEPAM GEL FILLED 20mg capsules \| \| d1ah. \| TEMAZEPAM GEL FILLED 15mg capsules \| \| d1ai. \| TEMAZEPAM GEL FILLED 30mg capsules \| \| d1aj. \| TEMAZEPAM 10mg/sachet oral solution \| \| d1ak. \| TEMAZEPAM 20mg/sachet oral solution \| \| d1al. \| EUHYPNOS 10mg/sachet oral solution \| \| d1am. \| EUHYPNOS 20mg/sachet oral solution \| \| d1an. \| EUHYPNOS 10mg/5mL oral solution \| \| d1ao. \| TEMAZEPAM 10mg/5mL sugar free oral solution \| \| d1b.. \| *TRIAZOLAM \| \| d1b1. \| TRIAZOLAM 125microgram tablets \| \| d1b2. \| TRIAZOLAM 250microgram tablets \| \| d1b3. \| *HALCION 125microgram tablets \| \| d1b4. \| *HALCION 250microgram tablets \| \| d1c.. \| TRICLOFOS SODIUM \| \| d1c1. \| *TRICLOFOS 500mg/5mL liquid \| \| d1d.. \| ZOPICLONE \| \| d1d1. \| ZOPICLONE 7.5mg tablets \| \| d1d2. \| ZIMOVANE 7.5mg tablets \| \| d1d3. \| ZOPICLONE 3.75mg tablets \| \| d1d4. \| ZIMOVANE LS 3.75mg tablets \| \| d1d5. \| *ZILEZE 7.5 tablets \| \| d1d6. \| *ZILEZE 3.75 tablets \| \| d1e.. \| *GLUTETHIMIDE [no drugs here] \| \| d1f.. \| ZOLPIDEM \| \| d1f1. \| STILNOCT 5mg tablets \| \| d1f2. \| ZOLPIDEM TARTRATE 5mg tablets \| \| d1f3. \| ZOLPIDEM TARTRATE 10mg tablets \| \| d1f4. \| STILNOCT 10mg tablets \| \| d1g.. \| ZALEPLON \| \| d1g1. \| SONATA 5mg capsules \| \| d1g2. \| SONATA 10mg capsules \| \| d1gy. \| ZALEPLON 5mg capsules \| \| d1gz. \| ZALEPLON 10mg capsules \| \| d1h.. \| MELATONIN \| \| d1h1. \| CIRCADIN 2mg m/r tablets \| \| d1hz. \| MELATONIN 2mg m/r tablets \| |
| **GP: ADHD** |
| \| 1P00. \| Hyperactive behaviour \| \| --- \| --- \| \| dc1.. \| DEXAMFETAMINE SULPHATE \| \| dc1w. \| DEXAMFETAMINE SULPHATE 5mg tablets \| \| dw1.. \| METHYLPHENIDATE \| \| dw11. \| METHYLPHENIDATE HYDROCHLORIDE 10mg tablets \| \| dw1q. \| METHYLPHENIDATE HYDROCHLORIDE 5mg m/r capsules \| \| dw1r. \| METHYLPHENIDATE HYDROCHLORIDE 27mg m/r tablets \| \| dw1s. \| METHYLPHENIDATE HYDROCHLORIDE 40mg m/r capsules \| \| dw1t. \| METHYLPHENIDATE HYDROCHLORIDE 10mg m/r capsules \| \| dw1u. \| METHYLPHENIDATE HYDROCHLORIDE 30mg m/r capsules \| \| dw1v. \| METHYLPHENIDATE HYDROCHLORIDE 36mg m/r tablets \| \| dw1w. \| METHYLPHENIDATE HYDROCHLORIDE 18mg m/r tablets \| \| dw1x. \| METHYLPHENIDATE HYDROCHLORIDE 20mg m/r capsules \| \| dw1y. \| METHYLPHENIDATE HYDROCHLORIDE 5mg tablets \| \| dw1z. \| METHYLPHENIDATE HYDROCHLORIDE 20mg tablets \| \| dw2.. \| ATOMOXETINE \| \| dw2u. \| ATOMOXETINE 80mg capsules \| \| dw2v. \| ATOMOXETINE 60mg capsules \| \| dw2w. \| ATOMOXETINE 40mg capsules \| \| dw2x. \| ATOMOXETINE 25mg capsules \| \| dw2y. \| ATOMOXETINE 18mg capsules \| \| dw2z. \| ATOMOXETINE 10mg capsules \| \| E.... \| Mental disorders \| \| E02y4 \| Drug-induced personality disorder \| \| E10.. \| Schizophrenic disorders \| \| E110. \| Manic disorder, single episode \| \| E110z \| Manic disorder, single episode NOS \| \| E2E.. \| Childhood hyperkinetic syndrome \| \| E2E0. \| Child attention deficit disorder \| \| E2E00 \| Attention deficit without hyperactivity \| \| E2E01 \| Attention deficit with hyperactivity \| \| E2E0z \| Child attention deficit disorder NOS \| \| E2E2. \| Hyperkinetic conduct disorder \| \| E2Ey. \| Other hyperkinetic manifestation \| \| E2Ez. \| Hyperkinetic syndrome NOS \| \| Eu90. \| [X]Hyperkinetic disorders \| \| Eu900 \| [X]Disturbance of activity and attention \| \| Eu901 \| [X]Hyperkinetic conduct disorder \| \| Eu902 \| [X]Deficits in attention, motor control and perception \| \| Eu90y \| [X]Other hyperkinetic disorders \| \| Eu90z \| [X]Hyperkinetic disorder, unspecified \| \| Eu9y7 \| [X]Attention deficit disorder \| |
| **GP: STIMULANT** |
| \| dw... \| DRUGS USED TO TREAT HYPERACTIVITY DISORDERS \| \| --- \| --- \| \| dw1.. \| METHYLPHENIDATE \| \| dw11. \| METHYLPHENIDATE HYDROCHLORIDE 10mg tablets \| \| dw12. \| RITALIN 10mg tablets \| \| dw13. \| *EQUASYM 5mg tablets \| \| dw14. \| *EQUASYM 20mg tablets \| \| dw15. \| *EQUASYM 10mg tablets \| \| dw16. \| EQUASYM XL 20mg m/r capsules \| \| dw17. \| CONCERTA XL 18mg m/r tablets \| \| dw18. \| CONCERTA XL 36mg m/r tablets \| \| dw19. \| *TRANQUILYN 5mg tablets \| \| dw1A. \| *TRANQUILYN 10mg tablets \| \| dw1B. \| *TRANQUILYN 20mg tablets \| \| dw1C. \| EQUASYM XL 10mg m/r capsules \| \| dw1D. \| EQUASYM XL 30mg m/r capsules \| \| dw1E. \| MEDIKINET XL 10mg m/r capsules \| \| dw1F. \| MEDIKINET XL 20mg m/r capsules \| \| dw1G. \| MEDIKINET XL 30mg m/r capsules \| \| dw1H. \| MEDIKINET XL 40mg m/r capsules \| \| dw1I. \| CONCERTA XL 27mg m/r tablets \| \| dw1J. \| MEDIKINET 5mg tablets \| \| dw1K. \| MEDIKINET 10mg tablets \| \| dw1L. \| MEDIKINET 20mg tablets \| \| dw1M. \| MEDIKINET XL 5mg m/r capsules \| \| dw1q. \| METHYLPHENIDATE HYDROCHLORIDE 5mg m/r capsules \| \| dw1r. \| METHYLPHENIDATE HYDROCHLORIDE 27mg m/r tablets \| \| dw1s. \| METHYLPHENIDATE HYDROCHLORIDE 40mg m/r capsules \| \| dw1t. \| METHYLPHENIDATE HYDROCHLORIDE 10mg m/r capsules \| \| dw1u. \| METHYLPHENIDATE HYDROCHLORIDE 30mg m/r capsules \| \| dw1v. \| METHYLPHENIDATE HYDROCHLORIDE 36mg m/r tablets \| \| dw1w. \| METHYLPHENIDATE HYDROCHLORIDE 18mg m/r tablets \| \| dw1x. \| METHYLPHENIDATE HYDROCHLORIDE 20mg m/r capsules \| \| dw1y. \| METHYLPHENIDATE HYDROCHLORIDE 5mg tablets \| \| dw1z. \| METHYLPHENIDATE HYDROCHLORIDE 20mg tablets \| \| dw2.. \| ATOMOXETINE \| \| dw21. \| STRATTERA 10mg capsules \| \| dw22. \| STRATTERA 18mg capsules \| \| dw23. \| STRATTERA 25mg capsules \| \| dw24. \| STRATTERA 40mg capsules \| \| dw25. \| STRATTERA 60mg capsules \| \| dw26. \| STRATTERA 80mg capsules \| \| dw2u. \| ATOMOXETINE 80mg capsules \| \| dw2v. \| ATOMOXETINE 60mg capsules \| \| dw2w. \| ATOMOXETINE 40mg capsules \| \| dw2x. \| ATOMOXETINE 25mg capsules \| \| dw2y. \| ATOMOXETINE 18mg capsules \| \| dw2z. \| ATOMOXETINE 10mg capsules \| \| E244. \| Amphetamine or other psychostimulant dependence \| \| E2440 \| Amphetamine or psychostimulant dependence, unspecified \| \| E2441 \| Amphetamine or psychostimulant dependence, continuous \| \| E2442 \| Amphetamine or psychostimulant dependence, episodic \| \| E2443 \| Amphetamine or psychostimulant dependence in remission \| \| E244z \| Amphetamine or psychostimulant dependence NOS \| \| E2570 \| Nondependent amphetamine/psychostimulant abuse, unspecified \| \| E2571 \| Nondependent amphetamine/psychostimulant abuse, continuous \| \| E2572 \| Nondependent amphetamine or psychostimulant abuse, episodic \| \| E2573 \| Nondependent amphetamine/psychostimulant abuse in remission \| \| E257z \| Nondependent amphetamine or psychostimulant abuse NOS \| \| Eu15. \| [X]Mental & behav disorder due other stimulants inc caffein \| \| SL97. \| Psychostimulant poisoning \| \| SL97z \| Psychostimulant poisoning NOS \| \| SLA.. \| Central nervous system stimulant poisoning \| \| SLAy. \| Other central nervous system stimulant poisoning \| \| SLAz. \| Central nervous system stimulant poisoning NOS \| \| T842. \| Accidental poisoning by psychostimulants \| \| T842z \| Accidental poisoning by psychostimulants NOS \| \| T843. \| Accidental poisoning by central nervous system stimulants \| \| T843z \| Accidental poisoning by central nervous system stimulant NOS \| \| TJ97. \| Adverse reaction to psychostimulants \| \| TJ97z \| Adverse reaction to psychostimulant NOS \| \| TJA.. \| Adverse reaction to central nervous system stimulants \| \| TJAy. \| Adverse reaction to other central nervous system stimulants \| \| TJAz. \| Adverse reaction to central nervous system stimulants NOS \| \| U60A. \| [X]CNS stimulants caus adverse effect in therapeut use, NEC \| \| U60A3 \| [X]Other CNS stimulants caus adverse effects in therap use \| \| U60A4 \| [X]Unspec CNS stimulants caus adverse effects in therap use \| \| U60D2 \| [X]Stimulant laxatives caus adverse effects in therap use \| |
| **GP: LEARNING DIFFICULTIES** |
| \| 918e. \| On learning disability register \| \| --- \| --- \| \| E3... \| Mental retardation \| \| E30.. \| Mild mental retardation, IQ in range 50-70 \| \| E31.. \| Other specified mental retardation \| \| E310. \| Moderate mental retardation, IQ in range 35-49 \| \| E311. \| Severe mental retardation, IQ in range 20-34 \| \| E312. \| Profound mental retardation with IQ less than 20 \| \| E31z. \| Other specified mental retardation NOS \| \| E3y.. \| Other specified mental retardation \| \| E3z.. \| Mental retardation NOS \| \| Eu7.. \| [X]Mental retardation \| \| Eu70. \| [X]Mild mental retardation \| \| Eu700 \| [X]Mld mental retard with statement no or min impairm behav \| \| Eu701 \| [X]Mld mental retard sig impairment behav req attent/treatmt \| \| Eu70y \| [X]Mild mental retardation, other impairments of behaviour \| \| Eu70z \| [X]Mild mental retardation without mention impairment behav \| \| Eu71. \| [X]Moderate mental retardation \| \| Eu710 \| [X]Mod mental retard with statement no or min impairm behav \| \| Eu711 \| [X]Mod mental retard sig impairment behav req attent/treatmt \| \| Eu71y \| [X]Mod retard oth behav impair \| \| Eu71z \| [X]Mod mental retardation without mention impairment behav \| \| Eu72. \| [X]Severe mental retardation \| \| Eu720 \| [X]Sev mental retard with statement no or min impairm behav \| \| Eu721 \| [X]Sev mental retard sig impairment behav req attent/treatmt \| \| Eu72y \| [X]Severe mental retardation, other impairments of behaviour \| \| Eu72z \| [X]Sev mental retardation without mention impairment behav \| \| Eu73. \| [X]Profound mental retardation \| \| Eu730 \| [X]Profound ment retrd wth statement no or min impairm behav \| \| Eu731 \| [X]Profound ment retard sig impairmnt behav req attent/treat \| \| Eu73y \| [X]Profound mental retardation, other impairments of behavr \| \| Eu73z \| [X]Prfnd mental retardation without mention impairment behav \| \| Eu7y. \| [X]Other mental retardation \| \| Eu7y0 \| [X]Oth mental retard with statement no or min impairm behav \| \| Eu7y1 \| [X]Oth mental retard sig impairment behav req attent/treatmt \| \| Eu7yy \| [X]Other mental retardation, other impairments of behaviour \| \| Eu7yz \| [X]Other mental retardation without mention impairment behav \| \| Eu7z. \| [X]Unspecified mental retardation \| \| Eu7z0 \| [X]Unsp mental retard with statement no or min impairm behav \| \| Eu7z1 \| [X]Unsp mentl retard sig impairment behav req attent/treatmt \| \| Eu7zy \| [X]Unspecified mental retardatn, other impairments of behav \| \| Eu7zz \| [X]Unsp mental retardation without mention impairment behav \| \| Eu814 \| [X]Moderate learning disability \| \| Eu815 \| [X]Severe learning disability \| \| Eu816 \| [X]Mild learning disability \| \| Eu817 \| [X]Profound learning disability \| \| Eu81z \| [X]Developmental disorder of scholastic skills, unspecified \| |
| **GP: CONDUCT DISORDER** |
| \| 1467 \| H/O: anorexia nervosa \| \| --- \| --- \| \| E140. \| Infantile autism \| \| E1400 \| Active infantile autism \| \| E140z \| Infantile autism NOS \| \| E2023 \| Social phobia, fear of eating in public \| \| E270. \| Stammering or stuttering \| \| E271. \| Anorexia nervosa \| \| E272. \| Tics \| \| E2722 \| Chronic motor tic disorder \| \| E2723 \| Gilles de la Tourette's disorder \| \| E2731 \| Head-banging \| \| E2747 \| Somnambulism - sleep walking \| \| E275. \| Other and unspecified non-organic eating disorders \| \| E2750 \| Unspecified non-organic eating disorder \| \| E2751 \| Bulimia (non-organic overeating) \| \| E2752 \| Pica \| \| E275y \| Other specified non-organic eating disorder \| \| E275z \| Non-organic eating disorder NOS \| \| E27z2 \| Lisping \| \| E27z4 \| Nail-biting \| \| E27z5 \| Thumb-sucking \| \| E2C.. \| Disturbance of conduct NEC \| \| E2C0. \| Aggressive unsocial conduct disorder \| \| E2C1. \| Nonaggressive unsocial conduct disorder \| \| E2C10 \| Unsocial childhood truancy \| \| E2C12 \| Tantrums \| \| E2C2. \| Socialised conduct disorder \| \| E2Cy0 \| Breath holder \| \| E2Cz. \| Unspecified disturbance of conduct \| \| E2Czz \| Disturbance of conduct NOS \| \| E2D22 \| Childhood and adolescent disturbance with elective mutism \| \| E2Dy0 \| Childhood and adolescent oppositional disorder \| \| E2E.. \| Childhood hyperkinetic syndrome \| \| E2E0. \| Child attention deficit disorder \| \| E2E00 \| Attention deficit without hyperactivity \| \| E2E01 \| Attention deficit with hyperactivity \| \| E2E0z \| Child attention deficit disorder NOS \| \| E2E2. \| Hyperkinetic conduct disorder \| \| E2Ez. \| Hyperkinetic syndrome NOS \| \| E2F.. \| Specific delays in development \| \| E2F0. \| Specific reading disorder \| \| E2F00 \| Reading disorder unspecified \| \| E2F02 \| Developmental dyslexia \| \| E2F1. \| Dyscalculia \| \| E2F2. \| Other specific learning difficulty \| \| E2F3. \| Speech or language developmental disorder \| \| E2F30 \| Developmental aphasia \| \| E2F3z \| Speech or language developmental disorder NOS \| \| E2F4. \| Coordination disorder (dyspraxia) \| \| E2F5. \| Mixed development disorder \| \| E2Fy. \| Other development delays \| \| E2Fz. \| Developmental disorder NOS \| \| E3... \| Mental retardation \| \| E30.. \| Mild mental retardation, IQ in range 50-70 \| \| E3z.. \| Mental retardation NOS \| \| Eu50. \| [X]Eating disorders \| \| Eu500 \| [X]Anorexia nervosa \| \| Eu501 \| [X]Atypical anorexia nervosa \| \| Eu502 \| [X]Bulimia nervosa \| \| Eu503 \| [X]Atypical bulimia nervosa \| \| Eu50z \| [X]Eating disorder, unspecified \| \| Eu63. \| [X]Habit and impulse disorders \| \| Eu80. \| [X]Specific developmental disorders of speech and language \| \| Eu800 \| [X]Specific speech articulation disorder \| \| Eu801 \| [X]Expressive language disorder \| \| Eu802 \| [X]Receptive language disorder \| \| Eu80y \| [X]Other developmental disorders of speech and language \| \| Eu80z \| [X]Developmental disorder of speech and language unspecified \| \| Eu81. \| [X]Specific developmental disorders of scholastic skills \| \| Eu810 \| [X]Specific reading disorder \| \| Eu81z \| [X]Developmental disorder of scholastic skills, unspecified \| \| Eu82. \| [X]Specific developmental disorder of motor function \| \| Eu83. \| [X]Mixed specific developmental disorders \| \| Eu84. \| [X]Pervasive developmental disorders \| \| Eu840 \| [X]Childhood autism \| \| Eu841 \| [X]Atypical autism \| \| Eu845 \| [X]Asperger's syndrome \| \| Eu84z \| [X]Pervasive developmental disorder, unspecified \| \| Eu9.. \| [X]Behavioural/emotional disords onset childhood/adolescence \| \| Eu900 \| [X]Disturbance of activity and attention \| \| Eu901 \| [X]Hyperkinetic conduct disorder \| \| Eu902 \| [X]Deficits in attention, motor control and perception \| \| Eu91. \| [X]Conduct disorders \| \| Eu912 \| [X]Socialized conduct disorder \| \| Eu913 \| [X]Oppositional defiant disorder \| \| Eu91z \| [X]Conduct disorder, unspecified \| \| Eu92. \| [X]Mixed disorders of conduct and emotions \| \| Eu940 \| [X]Elective mutism \| \| Eu941 \| [X]Reactive attachment disorder of childhood \| \| Eu953 \| [X]Involuntary excessive blinking \| \| Eu9y5 \| [X]Stuttering [stammering] \| \| Eu9y7 \| [X]Attention deficit disorder \| |
