# Supplementary material for: School achievement as a predictor of depression and self-harm in adolescence: linked education and health record study
Source: Br J Psychiatry. 2018 Mar 6;212(4):215–21. doi: 10.1192/bjp.2017.69 (PMC7557863; doi:10.1192/bjp.2017.69)
Supplement: Supplementary file 1 [file S0007125017000691sup.zip › S0007125017000691sup002.docx]

## Supplementary file 2: Hazard ratio for outcomes in adolescence - Adjusted (for ADHD and conduct disorder and learning difficulties) by gender.

|  | MALE: Depression after KS2  Hazard ratio (95%CI) n=9606 | FEMALE: Depression after KS2  Hazard ratio (95%CI) n=23,384 | MALE: Self-harm after KS2  Hazard ratio (95%CI) | FEMALE: Self-harm after KS2  Hazard ratio (95%CI) |
| --- | --- | --- | --- | --- |
| Not achieving at age 7 | 0.97 (0.87 to 1.09) | 0.91 (0.83 to 0.99) | 1.14 (0.95 to 1.36) | 1.18 (0.06 to 1.32) |
| Not achieving at age 11 | **1.26 (1.12 to 1.40)** | **1.26 (1.16 to 1.35)** | **1.23 (1.03 to 1.47)** | 0.97 (0.87 to 1.07) |
| Free school meals at age 11 | **1.38 (1.22 to 1.56)** | **1.47 (1.36 to 1.59)** | **1.39 (1.15 to 1.68)** | **1.58 (1.43 to 1.74)** |
| Free school meal at age 7 | **1.41 (1.25 to 1.59)** | **1.33 (1.23 to 1.44)** | **1.92 (1.60 to 2.31)** | **1.54 (1.4 to 2.71)** |
| ADHD (in primary school) | 1.16 (0.73 to 1.67) | 1.2 (0.7 to 2.08) | **1.50 (1.84 to 2.68)** | 1.61 (0.92 to 2.8) |
| Conduct disorder (in primary school) | **1.80 (1.45 to 1.25)** | **1.31 (1.01 to 1.70)** | **1.62 (1.14 to 2.30)** | **1.72 (1.29 to 2.29)** |
| Learning difficulties (in primary school) | 0.52 (0.19 to 1.41) | 0.15 (0.02 to 1.12) | 0.55 (0.13 to 2.24) | 0.38 (0.95 to 1.58) |
| Hypnotic prescription (primary school) | **1.72 (1.01 to 2.91)** | 0.83 (0.38 to 1.79) | 1.24 (0.61 to 2.5) | 0.94 (0.43 to 2.03) |
| Stimulant prescription (in primary school) | 0.79 (0.48 to 1.29) | 1.13 (0.58 to 2.20) | 1.4 (0.77 to 2.57) | 1.13 (0.58 to 2.18) |

Subsets:

|  | MALE: Depression after KS3 (age 14-20) | FEMALE: Depression after KS3 (age 14 to 20) | Self harm after KS3 (age 14-20) | Self harm before KS3 (age 12 to 14) |
| --- | --- | --- | --- | --- |
| Not achieving at age 14 | **1.81 (1.44 to 2.28)** | **1.41 (1.29 to 1.53)** | **1.81(1.44 to 2.28)** | **1.55 (1.37 to 1.75)** |
|  | Depression after KS4 (age 16-20) | Depression before KS4 (age 12 to 16) | Self harm after KS4 (age 16-20) | Self harm before KS4 (age 12 to 16) |
| Not achieving at age 16 | **1.88 (1.07 to 3.28)** | **1.55 (1.31 to 1.83)** | **1.88 (1.07 to3.28)** | **1.51 (1.06 to 2.14)** |

*adjusted for KS1, KS2, FSM and Female gender
